# Pain and the biochemistry of fibromyalgia: patterns of peripheral cytokines and chemokines contribute to the differentiation between fibromyalgia and controls and are associated with pain, fat infiltration and content

**DOI:** 10.3389/fpain.2024.1288024

**Published:** 2024-01-18

**Authors:** Björn Gerdle, Olof Dahlqvist Leinhard, Eva Lund, Peter Lundberg, Mikael Fredrik Forsgren, Bijar Ghafouri

**Affiliations:** ^1^ Pain and Rehabilitation Centre, and Department of Health, Medicine and Caring Sciences, Linköping University, Linköping, Sweden; ^2^ Center for Medical Image Science and Visualization (CMIV), Linköping, Sweden; ^3^ Radiation Physics, Department of Health, Medicine and Caring Sciences, Linköping University, Linköping, Sweden; ^4^ AMRA Medical AB, Linköping, Sweden; ^5^ Department of Radiation Physics, and Department of Health, Medicine and Caring Sciences, Linköping University, Linköping, Sweden

**Keywords:** adipose, biomarker, body composition, fat, fibromyalgia, inflammation, immune, pain

## Abstract

**Objectives:**

This explorative study analyses interrelationships between peripheral compounds in saliva, plasma, and muscles together with body composition variables in healthy subjects and in fibromyalgia patients (FM). There is a need to better understand the extent cytokines and chemokines are associated with body composition and which cytokines and chemokines differentiate FM from healthy controls.

**Methods:**

Here, 32 female FM patients and 30 age-matched female healthy controls underwent a clinical examination that included blood sample, saliva samples, and pain threshold tests. In addition, the subjects completed a health questionnaire. From these blood and saliva samples, a panel of 68 mainly cytokines and chemokines were determined. Microdialysis of trapezius and erector spinae muscles, phosphorus-31 magnetic resonance spectroscopy of erector spinae muscle, and whole-body magnetic resonance imaging for determination of body composition (BC)—i.e., muscle volume, fat content and infiltration—were also performed.

**Results:**

After standardizing BC measurements to remove the confounding effect of Body Mass Index, fat infiltration and content are generally increased, and fat-free muscle volume is decreased in FM. Mainly saliva proteins differentiated FM from controls. When including all investigated compounds and BC variables, fat infiltration and content variables were most important, followed by muscle compounds and cytokines and chemokines from saliva and plasma. Various plasma proteins correlated positively with pain intensity in FM and negatively with pain thresholds in all subjects taken together. A mix of increased plasma cytokines and chemokines correlated with an index covering fat infiltration and content in different tissues. When muscle compounds were included in the analysis, several of these were identified as the most important regressors, although many plasma and saliva proteins remained significant.

**Discussion:**

Peripheral factors were important for group differentiation between FM and controls. In saliva (but not plasma), cytokines and chemokines were significantly associated with group membership as saliva compounds were increased in FM. The importance of peripheral factors for group differentiation increased when muscle compounds and body composition variables were also included. Plasma proteins were important for pain intensity and sensitivity. Cytokines and chemokines mainly from plasma were also significantly and positively associated with a fat infiltration and content index.

**Conclusion:**

Our findings of associations between cytokines and chemokines and fat infiltration and content in different tissues confirm that inflammation and immune factors are secreted from adipose tissue. FM is clearly characterized by complex interactions between peripheral tissues and the peripheral and central nervous systems, including nociceptive, immune, and neuroendocrine processes.

## 1 Introduction

Fibromyalgia (FM), which has a prevalence of 1%–4% in the population and a female predominance, is a complex chronic pain condition characterized by generalized widespread pain and hyperalgesia/allodynia (1–4). Psychological distress, fatigue, cognitive problems, inflammatory bowel disease, and insomnia are frequent symptoms and comorbidities. Modern management of chronic pain conditions rely on a biopsychosocial approach. However, mechanism-based diagnoses are lacking in the chronic pain field. Interventions that do not target causal mechanisms (e.g., molecular mechanisms) but modulate symptoms and risk factors are associated with high numbers needed to treat (NNT) (5). The International Association for the Study of Pain (IASP) in 2017 added to the existing mechanistic pain descriptors, nociceptive pain and neuropathic pain, a new mechanistic descriptor—nociplastic pain; FM was considered as a nociplastic pain condition (6).

Internationally agreed central or peripheral biomarkers are lacking for the International Classification of Diseases (ICD) diagnosis of FM, which partially may be because no definite pathophysiology for FM has been established. Alterations in the central and peripheral nervous systems as well as peripheral tissues have been reported in FM (7–18); central alterations are clinically recognized. Investigating peripheral and systemic alterations are now given increased attention. Hence, signs of mitochondrial dysfunction from muscles of patients with FM have been reported [e.g., increased muscle interstitial lactate, pyruvate and/or glutamate levels (19–22), decreased muscle concentrations of adenosine triphosphate (ATP) or nucleoside triphosphates (NTP) and phosphocreatine (PCr) (19, 23–25)], and alterations in metabolism of N-acylethanolamines (26, 27), and high prevalence of small fibre pathology (18, 28). Moreover, altered gut microbiome composition has been reported in FM (29, 30). Systemic alterations have also been found e.g., significant alterations in lipid mediators (31, 32). Blood proteomic studies (generally proteins at nano- and micromolar levels) show significant signs of immunity and inflammation alterations, which may indicate systemic low-grade inflammation (28, 33–36). However, the roles of commonly investigated blood cytokines and chemokines (typically found at picomolar levels) are unclear. Three recent systematic reviews of FM are not in consensus about which cytokines are significantly altered, except for IL-6 (37–39).

There are several indications that widespread pain including FM, obesity and low-grade inflammation are interrelated. Hence, obesity is a risk factor for FM development and is prevalent in this pain condition (approx. 36%) (40, 41). Severe clinical presentation and worse outcomes in pharmacotherapy are found in FM patients with obesity (28, 42–44), but it is unclear whether obesity is a causal factor or a result of the condition. Several studies indicate that obesity has an inflammatory component and is associated with chronic low-grade inflammation in, for example, adipose tissue, skeletal muscle, liver, pancreas, intestine and brain (45). In obese subjects, visceral and abdominal fat tissues secrete, for example, cytokines, chemokines (e.g., TNF-α, IL-6, IL-18, MCP-1/CCL2, CCL19, HGF, CSF-1, and VEGF-A), osteopontin, interferon-gamma, adipokines and micro-RNAs that can exert functional and pathophysiological alterations in various tissues (46–49). However, the knowledge concerning such secretions in chronic widespread pain including FM is lacking and the number of cytokines and chemokines per study is generally limited. Moreover, their influence on the clinical presentation is not understood. Body Mass Index (BMI), traditionally used to indicate overweight and obesity, has several disadvantages compared to more quantitative organ specific variables indicating fat content and infiltration. Recently, we reported significantly increased fat infiltration and content in different organs in this FM cohort (28). As there is a strong link between adipose tissue and BMI (28), there is a need to standardize such body composition variables (i.e., removing the association to BMI) to increase the knowledge about fat infiltration and content in FM.

Thus, there is a knowledge gap concerning the relationship between body composition (abdominal adipose tissue volumes and fat infiltration as well as muscle volumes and fat infiltration) and cytokines, chemokines and growth factors in FM compared to healthy controls. This study is part of a major project investigating mainly peripheral and systemic alterations in FM. In addition to finding that this FM cohort had increased fat infiltration and content in different organs, we found that this FM cohort had muscle alterations (i.e., increased pyruvate levels and lowered levels of ATP and PCr) (19, 28). Data from these studies are included for a broad coverage of peripheral factors.

Thus, this explorative study has two main aims:

I. To study whether FM patients have different profiles regarding inflammatory proteins, metabolites, markers of muscle energetic system, fat infiltration and body composition.
II. To investigate whether there are significant associations between the specific profiles of biochemical compounds and clinical variables (pain intensity, pressure pain thresholds, psychological distress, and health).

## 2 Subjects and methods

### 2.1 Subjects

This study includes 32 female FM patients and 30 age-matched female controls (CON). The recruitment process and sample size calculations have been presented elsewhere (19). Brief descriptions of inclusion and exclusion criteria as well as the clinical examinations are given below.

The Regional Ethical Review Board in Linköping (Dnr: 2016/239-31) approved the study. All participants gave their written informed consent, and the study was performed in accordance with the Helsinki Declaration.

### 2.2 Procedures

At the first visit, the subjects underwent a clinical examination that included collecting blood and saliva samples, registrations of pain thresholds. In addition, the subjects completed a health questionnaire. Microdialysis of the interstitium of the trapezius and erector spinae muscles, phosphorus-31 magnetic resonance spectroscopy (P-31 MRS) of the erector spinae muscle, and whole-body magnetic resonance imaging were performed during other visits.

### 2.3 Questionnaire

The questionnaire captured age (years) and aspects of pain, psychological distress, and health. Pain aspects were determined using a numeric rating scale (0 = no pain and 10 = worst possible pain) and the global pain intensity for the previous seven days was indicated (NRS-7d). The duration of FM (years) was also reported. Psychological distress was determined using the Hospital Anxiety and Depression Scale (HADS) (0–42) (50–52). A lower score indicates fewer psychological distress symptoms.

Health aspects were determined using the European Quality of Life Instrument (EQ-5D). The ED-D has two parts. One captures a patient's perceived state of health, and one is a self-estimation of today's health on a 100-point scale (EQ5D-VAS) where low values indicate low perceived health (53, 54). This study uses the latter.

### 2.4 Clinical examinations

As the clinical examination used has been described in detail elsewhere, here we provide a brief description (19). Both patients and controls underwent a brief clinical examination of heart and lungs, which included recording diastolic and systolic blood pressure (mm Hg) after 2 min of rest in the horizontal position. In addition, their weight (kg) and height (m) were recorded. Body Mass Index (BMI) (kg/m^2^) was calculated. The clinical examination also ensured that the controls were healthy with respect to anamnesis for rheumatic diseases, neurological diseases, diabetes, cardio-vascular diseases, psychiatric diseases, and high alcohol consumption. The clinical examination of the patients ensured that they met the American College of Rheumatology (ACR) 1990 criteria (1), although newer FM criteria (2010/2011 and 2016) have been presented (55–57). However, we wanted to compare our study with earlier studies, so we chose the 1990 ACR criteria. Both in the FM and in the CON the number of tender points was registered as an indicator of hyperalgesia/allodynia.

### 2.5 Pressure pain thresholds

The measurements of pressure pain thresholds (PPT) have been described in detail elsewhere (58, 59). PPT were determined using a manual pressure algometer (contact area of 1 cm^2^; pressure was increased by 30 kPa/s) (Somedic AB, Sweden). The erector spinae, tibialis anterior, and trapezius were investigated bilaterally. The subject-perceived pain was indicated by pushing a stop button or until the maximum threshold of 600 kPa was reached. At each site, two measurements were made with a minimum interval of 30 s. The mean of the six anatomical locations (PPT-tot) is reported as a global measure of pain sensitivity (i.e., low values indicate hyperalgesia).

### 2.6 Saliva and blood samples

Subjects were instructed not to smoke, drink caffeine, eat, or brush their teeth 1 h before sampling and to rest, preferably lying down, 30 min before each sampling. Saliva samples were collected using Salivette tubes (Sarstedt, Landskrona, Sweden). Samples were stored at 20 °C until they were assayed. Venous blood samples were collected in two 8-ml EDTA tubes. The samples were centrifuged at 1,000×*g* for 15 min, and the separate layers of plasma from the two blood samples, approximately 5–6 ml in total, were collected into a 12 ml Falcon-tube and mixed gently. The plasma was aliquoted into small portions and stored in −86 °C until analysis.

#### 2.6.1 Chemical analyses

Analyses of inflammatory proteins (cytokines, chemokines, and growth factors) were performed directly in plasma and saliva using the multiplex immunoassay technology Meso Scale (MSD) (Maryland, USA). Using the manufacturer's protocols for MSD, we analysed up to 71 custom kit-panel inflammatory substances with the electro-chemo-luminescence method. Data were collected and analysed using MESO QUICKPLEX SQ 120 instrument equipped with DISCOVERY WORKBENCH® data analysis software. On the day of analysis, samples were thawed, and randomly mixed. An 8-points standard curve with known concentrations of each protein was performed in duplicate and samples were run in single analysis. The CV for the analytes together with the lower and upper limits of detection (LLOD-ULOD), expressed in pg/ml, for each substance is presented in Supplementary Table 1. Proteins not detected in at least 50% of the samples in either patients or healthy controls were excluded from the statistical analysis. Therefore 68 proteins were included in the data analysis.

### 2.7 Whole-body magnetic resonance imaging

#### 2.7.1 MR measurements

Body composition was determined by quantitative analysis of magnetic resonance imaging (MRI). Details have been reported elsewhere (28, 60–64). In brief, subjects were scanned in a Philips Ingenia 3T MRI scanner (Philips Healthcare, Best, The Netherlands) using a 6-min dual-echo Dixon protocol, providing water and fat separated volumetric data covering a region from the neck to the knees (60).

#### 2.7.2 Profiling variables

The body composition profiling was performed using AMRA® Researcher (AMRA Medical AB, Linköping, Sweden) (65). The following variables were obtained for this study: visceral adipose tissue volume [VAT (L)]; abdominal subcutaneous adipose tissue volume [ASAT (L)]; liver fat by proton density fat-fraction [LF (%)]; total thigh fat-tissue free muscle volume (FFMV) of the thigh muscles [T-FFMV (L)]; and erector spinae [ES-FFMV (L)] muscles and mean muscle fat infiltration [MFI (%)] of the anterior thigh (T-MFI) and erector spinae (ES-MFI) muscles. These variables are not standardized (see below) and are summarized below as non-standardized body composition variables. The spinal erector group, a region between the top of first lumbar vertebrae (L1) and the bottom of the fifth lumbar vertebrae (L5), includes the iliocostalis, longissimus, spinalis, and the transversospinales as this small muscle group cannot be excluded from the spinal erector group given the spatial resolution of the image data.

#### 2.7.3 Body composition measurement standardization

The body composition measurements were standardized (i.e., not normalized) in the manner described below resulting in body composition standard scores (commonly referred to as *z*-scores) using individualized virtual control groups (VCG) obtained from a very large reference population. The procedure results in a standardization of a subject's body composition measurements by subtracting the reference VCG mean from the subject's measurement and then dividing the difference by the reference VCG standard deviation. As this VCG is based on sex and BMI matching to the target subject, the standardized body composition measurements (*z*-scores) following this operation are effectively no longer confounded by sex and BMI. Non-standardized body composition data are also presented as a comparison.

#### 2.7.4 Detailed procedure

Using data from a sample of 40,178 participants of a normal reference population scanned in the UK Biobank imaging study (66, 67), we calculated standardized body composition variables (i.e., *z*-scores, which are summarized as the standardized body composition variables below). Specifically, for each subject, an ASAT *z*-score (*z*-ASAT), VAT *z*-score (*z*-VAT), LF *z*-score (*z*-LF), T-MFI *z*-score (*z*-T-MFI), and T-FFMV *z*-score (*z*-T-FFMV) were calculated using the distribution of matched VCG. To create a VCG, we matched the target study subject to participants in the UK Biobank with the same sex and within ±1 kg/m^2^ of their BMI (except for T-FFMV, which was within ±2 kg/m^2^). If fewer than 150 controls were stratified by these criteria, the BMI interval was incrementally and symmetrically increased by 0.1 kg/m^2^ until the VCG contained at least 150 subjects.

Each subject's body compartment *z*-scores were calculated as the number of standard deviations between the VCG mean and study participants' body compartments measurement. In other words, the *z*-score measures how much the study subject deviates for each specific body compartment—i.e., from what is expected by their sex and BMI. VAT, ASAT, and FFMV values were divided by height squared (*h*^2^) before calculation of the *z*-score. Because of the observed skewed distribution of liver fat, LF values were log transformed before calculating *z*-LF. MFI was used without any transformation (68–70). We label the variables from the whole-body magnetic resonance imaging body composition (BC) variables. Specifically, BMI will not be included in the multivariate analyses as it is not a formal measure of body composition.

### 2.8 Data from microdialysis and magnetic resonance

This study also includes some data presented elsewhere obtained from microdialysis of the interstitium of trapezius and erector spinae muscles and from phosphorus-31 magnetic resonance spectroscopy (P-31 MRS) of the erector spinae muscle (19). Hence, from the microdialysis, we include concentrations of lactate, pyruvate, and glutamate immediately after the trauma period—i.e., at baseline (denoted as 140 min)—and the mean value of the time points from baseline to the end of the recovery (140–220 min). From P-31 MRS, the concentrations of nucleotide triphosphate (NTP, mainly composed of ATP), phosphocreatine (PCr), and inorganic phosphate (Pi) together with tissue pH (derived from the P-31 MRS measurements) were included in this study.

### 2.9 Statistics

The statistics were performed using the statistical packages IBM SPSS Statistics (version 27.0; IBM Corporation, Route 100 Somers, New York, USA) and SIMCA-P+ (version 17.0; Sartorius Stedim Biotech, Umeå, Sweden). A *P*-value of <0.05 was considered statistically significant. Text and tables report the mean value ± one standard deviation (±1 SD) of continuous variables, and percentages (%) are reported for categorical variables. To compare groups, we used Student's *t*-test for un-paired observations and Chi square test for proportions.

Previous studies have discussed the necessity of using advanced multivariate analyses (MVDA) when accounting for system-wide aspects, including missing data and multicollinearity problems (71, 72). Using SIMCA-P+, we applied advanced Principal Component Analysis (PCA) to determine multivariate outliers and multivariate correlation patterns. Outliers were identified using two methods: score plots in combination with Hotelling's *T*^2^ and distance to model in X-space. PCA extracts and displays systematic variation in the data matrix (i.e., a variant of multivariate correlation analysis). A cross validation technique was used to identify nontrivial components [p]. Variables loading on the same component [p] were correlated, and variables with high loadings but opposing signs were negatively correlated. Variables with high absolute loadings were considered significant. Per definition, the obtained components are not correlated and are arranged in decreasing order with respect to explained variation. Goodness of fit (*R*^2^) is the fraction of sum of squares of all the variables explained by a principal component (73). *Q*^2^ describes the goodness of prediction—the fraction of the total variation of the variables that can be predicted using principal component cross validation methods (73).

To determine group associations, we used Orthogonal Partial Least Square Regressions discriminant analysis (OPLS-DA). Orthogonal Partial Least Square Regressions (OPLS) was used for the multivariate regression analyses of the indices indicating fat infiltration and content and fat free muscle volume. SIMCA-P+ uses the Non-linear Iterative Partial Least Squares (NIPALS) algorithm to handle missing data: max 50% missing data for variables/scales and max 50% missing data for subjects. The Variable Influence on Projection (VIP) indicates the relevance of each *X*-variable pooled over all dimensions and *Y*-variables—i.e., the group of variables that best explains Y (73). VIP >1.0 (or VIPpred if more than one component is identified) was considered significant if VIP had 95% jack-knife uncertainty confidence interval non-equal to zero. *P*(corr) was used to note the direction of the relationship (positive or negative)—i.e., the loading of each variable was scaled as a correlation coefficient and therefore standardized the range from −1 to +1 (71). *P*(corr) is typically stable during iterative variable selection and comparable between models. Thus, a variable/regressor was considered statistically significant when VIP or VIPpred >1.0. If certain predefined internal criteria of SIMCA-P are fulfilled, a regression model will be obtained, including one or several components (the first is always the predictive component). The validity of the model is then determined using cross validation.

Hence, for each regression, we report *R*^2^, *Q*^2^, and the *P*-value of a cross-validated analysis of variance (CV-ANOVA). When more than 50 regressors were included, the OPLS analysis was made in two steps. In the first step, all relevant compounds were included in the analysis. In the second step, the compounds with VIP or VIPpred >1.5 were used in a new OPLS-DA or OPLS provided that the first analysis resulted in one or several significant components according to the internal rules used in SIMCA-P+ (73). The results of the second (final) regression are presented in text and tables including *R*^2^, *Q*^2^, and *P*-value of CV-ANOVA. The parameters of the initial regression are given in Supplementary Table 2.

## 3 Results

### 3.1 Descriptive data

Data obtained from the questionnaires, the clinical examinations, and the body composition (BC) variables (except the standardized variables) for healthy controls (CON) and in FM patients have been presented elsewhere (Table 1) (19, 28). FM had lower PPT and reported lower perceived health. The FM group reported medium pain intensity on the group level. As previously reported, pyruvate levels in trapezius and erector spinae were significantly higher, but NTP and PCr of erector spinae were significantly lower in FM (Table 1) (19). We recently reported that BMI and all non-standardized fat infiltration and content variables as well as T-FFMV differed significantly between the two groups (Table 1) (28). The group differences were more pronounced for the non-standardized BC variables than for the standardized (Table 1). Following standardization (i.e., correcting for BMI differences), all standardized BC variables except *z*-LF remained significantly different between the two groups—i.e., the differences in these BC variables between groups cannot be explained by the group difference in BMI.

**Table 1:** Data obtained from questionnaires, the clinical examinations, the body composition variables, and variables from muscle microdialysis and spectroscopy in controls (CON) and in fibromyalgia patients (FM) [mean and standard deviation (SD)].

Group	CON	*N* = 30	FM	*N* = 32	Statistics
Variables	Mean	SD	Mean	SD	*P*
Questionnaires and clinical examinations					
Age	42.80	9.88	40.00	11.23	n.s.
PPT-tot	385.01	111.53	138.24	86.76	<0.001*
FM duration (years)	NA	NA	5.63	5.76	NA
NRS-7d	0.00	0.00	6.54	1.69	<0.001*
HADS	4.23	3.65	13.32	6.11	<0.001*
EQ5D-VAS	86.90	7.57	51.86	18.84	<0.001*
Body composition variables					
BMI (kg/m^2^)	23.98	3.07	29.15	6.07	<0.001*
VAT (L)	1.272	0.803	3.130	2.017	<0.001*
ASAT (L)	6.420	2.957	11.101	5.494	<0.001*
LF (%)	0.018	0.009	0.055	0.072	0.010*
T-MFI (%)	0.059	0.012	0.075	0.019	<0.001*
ES-MFI (%)	0.080	0.023	0.104	0.036	0.003*
T-FFMV (L)	10.111	0.945	9.336	1.503	0.021*
ES-FFMV (L)	0.634	0.082	0.623	0.153	n.s.
*z*-VAT (SD)	−1.203	0.597	−0.538	0.943	0.002*
*z*-ASAT (SD)	−0.618	1.254	0.190	1.353	0.019*
*z*-LF (SD)	0.718	0.934	0.863	1.217	n.s.
*z*-T-MFI (SD)	−1.058	0.705	−0.583	0.804	0.018*
*z*-T-FFMV (SD)	1.706	0.773	0.479	1.272	<0.001*
Muscle Microdialysis variables					
Lactate Trap 140 min (mmol L^−1^)	2.55	1.62	2.71	1.67	n.s.
Lactate Trap 140–220 min (mmol L^−1^)	3.51	2.33	3.22	1.48	n.s.
Lactate ES 140 min (mmol L^−1^)	1.86	1.04	2.68	3.80	n.s.
Lactate ES 140–220 min (mmol L^−1^)	2.23	1.36	3.65	5.58	n.s.
Pyruvate Trap 140 min (μmol L^−1^)	12.97	10.98	27.96	18.19	<0.001*
Pyruvate Trap 140–220 min (μmol L^−1^)	20.10	17.87	38.64	29.75	0.005*
Pyruvate ES 140 min (µmol L^−1^)	12.37	10.97	26.59	30.74	0.032*
Pyruvate ES 140–220 min (µmol L^−1^)	18.57	15.97	44.15	71.35	0.079
Glutamate Trap 140 min (mmol L^−1^)	49.77	31.65	56.06	32.50	n.s.
Glutamate Trap 140–220 min (mmol L^−1^)	63.24	30.87	67.55	34.33	n.s.
Glutamate ES 140 min (mmol L^−1^)	41.40	26.52	30.20	43.02	n.s.
Glutamate ES 140–220 min (mmol L^−1^)	41.79	26.23	33.82	39.23	n.s.
Muscle Spectroscopy variables					
PCr (mM)	40.06	8.54	34.07	11.49	0.024*
Pi (mM)	5.68	1.84	5.29	1.76	n.s.
NTP (mM)	8.64	1.49	7.57	1.91	0.017*
pH	7.03	0.03	7.03	0.03	n.s.

CON, controls; FM, fibromyalgia; N, number of subjects; SD, standard deviation; PPT-tot, Pressure Pain Threshold, mean of six sites; NRS-7d, global pain intensity previous 7 days using a numeric rating scale (NRS); HADS, The Hospital Anxiety and Depression Scale; EQ5D-VAS, Perceived Health according to European Quality of Life (EQ); NA, Not Applicable; BMI, Body Mass Index; VAT, Visceral Adipose Tissue volume; ASAT, Abdominal Subcutaneous Adipose Tissue volume; LF, liver fat; T-MFI, Thigh Muscle Fat Infiltration; ES, erector spinae; Trap, trapezius muscle; ES-MFI, Erector Spinae Muscle Fat Infiltration; T-FFMV, Thigh Fat-free Muscle Volume; ES-FFMV, Erector Spinae Fat-free Muscle Volume; *z*-, standardized body composition variable. The concentrations of lactate, pyruvate and glutamate are presented levels immediately after the trauma period—i.e., at baseline (denoted as 140 min) and the mean value of the time points from baseline to the end of the recovery (140–220 min). PCr, phosphocreatine; Pi, inorganic Phosphate; NTP, nucleotide triphosphate (NTP, mainly composed of adenosine triphosphate ATP).

On the far right are the results of group comparisons (*P*-value). These results (except for the standardized body composition variables) have been published elsewhere (19, 28)].

* Denotes significant group difference.

### 3.2 Interrelationships between the body composition variables

The interrelationships between all BC variables were investigated, resulting in a PCA model with two significant components: *R*^2^(cumulative) = 0.59 and *Q*^2^(cumulative) = 0.20. One patient (no. 16) was a multivariate outlier and therefore omitted from the analyses. Also, in the final analysis, two significant components were obtained (Supplementary Table 3). The first and thereby the most important component *p*[1] was dominated by body fat content and muscle infiltration variables: VAT, T-MFI, ASAT, ES-MFI, *z*-VAT and *z*-T-MFI. The second component *p*[2] mainly reflected the intercorrelations between the three fat free muscle volume variables: T-FFMV, ES-FFMV and *z*-T-FFMV.

We also performed separate PCA analyses for the non-standardized and the standardized BC variables. No significant model was achieved for the PCA of the standardized BC variables. For the non-standardized body composition variables, a significant PCA with two components was obtained: *R*^2^(cumulative) = 0.70 and *Q*^2^(cumulative) = 0.28. Fat infiltration and content variables—which were all positively intercorrelated—mainly determined the first component and the fat free muscle volume variables were important for the second component (Supplementary Table 4). The scores of the first component were defined as a fat infiltration and content index (FIC-index), and the scores of the second component were labelled Muscle-index. These two indices are per definition un-correlated. Significant group differences were noted for both indices: FIC-index: CON = −0.91 ± 1.01 vs. FM = 0.85 ± 2.02, *P* < 0.001; Muscle-index: CON = 0.46 ± 0.85 vs. FM = −0.43 ± 1.43, *P *= 0.005. Thus, the higher FIC scores in FM than in CON indicate more adipose tissue, whereas higher Muscle-index scores indicate larger fat-free muscle volume in CON than in FM.

### 3.3 Regression of group membership (CON or FM)

#### 3.3.1 Group differentiation using BC variables

To understand the relative importance of the BC variables in relation to group membership, we performed an OPLS-DA using the non-standardized and the standardized BC variables as regressors. For both regressions, significant models of group membership were obtained. Hence, both these regressions included intramuscular fat infiltration of the thigh and visceral adipose tissue volume as significant regressors. However, in the regression using the standardized BC variables, the fat free muscle volume of the thigh was the most important significant variable.

Specifically, the significant OPLS regression using the non-standardized BC variables as regressors (*R*^2 ^= 0.40, *Q*^2 ^= 0.33, CV-ANOVA *P*-value = 0.00014, one predictive and one orthogonal component) identified the following variables as significant: T-MFI [VIP = 1.32, *p*(corr) = 0.80], VAT [VIP = 1.28, *p*(corr) = 0.77], and ASAT [VIP = 1.10, *p*(corr) = 0.67]. The corresponding significant analysis using the standardized BC variables (*R*^2 ^= 0.26, *Q*^2 ^= 0.20, CV-ANOVA *P*-value = 0.0017, one predictive component) identified the following variables as significant: *z*-T-FFMV [VIP = 1.29, *p*(corr) = −0.86], *z*-T-MFI [VIP = 1.07, *p*(corr) = 0.71], and *z*-VAT [VIP = 1.03, *p*(corr) = 0.69].

#### 3.3.2 Group differentiation using compounds from saliva, plasma and/or muscles

The first regression included all compounds from saliva, plasma, and muscles as regressors (X variables). Certain saliva proteins and one microdialysis variable were particularly important significant regressors as shown in Table 2 (*R*^2 ^= 0.36, *Q*^2 ^= 0.22, CV-ANOVA *P*-value = 0.0165, one predictive component and one orthogonal component). The most significant saliva proteins—MIP-1α/CCl3, I-TAC/CXCL11, MCP-4/CCL13, MIP-3β/CCL19, and IL-2—showed positive associations with the group and therefore were increased in FM (Table 2). As expected from this regression, after including only saliva proteins as regressors, a significant model was also obtained. The significant proteins (*R*^2 ^= 0.18, *Q*^2 ^= 0.14, CV-ANOVA *P*-value = 0.019, one predictive component) are shown in Supplementary Table 5. The most important significant saliva proteins were identical to those presented in Table 2. As it was not possible to significantly differentiate the two groups of subjects using only plasma proteins as regressors, a combination with other data was required.

**Table 2:** OPLS-DA of group membership (FM denoted 1 and CON denoted 0) using compounds from saliva, plasma, and muscles as regressors. Variables with VIP > 1.5 are shown.

Variables	VIPpred	*p*(corr)
S-MIP-1α/CCL3	2.19	0.81
S-I-TAC/CXCL11	1.99	0.76
S-MCP-4/CCL13	1.98	0.75
S-MIP-3β/CCL19	1.87	0.71
S-IL-2	1.83	0.70
S-MDC/CCL22	1.80	0.67
S-CTACK/CCL27	1.80	0.69
S-G-CSF	1.78	0.67
S-MIP-3α/CCL20	1.77	0.67
S-MIP-5/CCL15	1.75	0.65
S-Eotaxin-2/CCL24	1.73	0.65
S-IL-5	1.71	0.65
S-SDF-1alpha/CXCL12	1.70	0.65
S-IL-12p70	1.65	0.63
M-pyruvate −140 to 220 min-Tr	1.60	0.58
S-GRO-alpha/CXCL1	1.60	0.61
S-IL-16	1.60	0.60
S-IL-17A	1.59	0.61
S-IL-13	1.58	0.59
S-TARC/CCL17	1.57	0.60
S-IL-17A-F	1.57	0.59
S-IL-10	1.55	0.60
S-IL-27	1.54	0.56
S-TNF-β/LTA	1.51	0.57
S-IFN-α2a	1.50	0.53
*R* ^2^	0.36	
*Q* ^2^	0.22	
CV-ANOVA *P*-value	0.0165	

VIPpred and *p*(corr) are reported for each regressor (i.e., the loading of each variable scaled as a correlation coefficient and therefore standardizing the range from −1 to +1). The sign of *p*(corr) indicates the direction of the correlation with the dependent variable (+ = positive correlation, i.e., higher in FM; − = negative correlation, i.e., lower in FM). The three bottom rows report *R*^2^, *Q*^2^, and *P*-value of the CV-ANOVA. In variable names: P, plasma; S, saliva; M, muscle. For protein names, see Supplementary Table 1. Tr: trapezius (M-pyruvate −140 to 220 min); Tr: mean value of pyruvate in the trapezius for the time points from baseline to the end of the recovery (140–220 min).

Using the muscle compounds obtained from microdialysis and MR spectroscopy when regressing group membership also resulted in a significant model (*R*^2 ^= 0.28, *Q*^2 ^= 0.16, CV-ANOVA *P*-value = 0.009; one predictive component). The following significant regressors were identified: Pyruvate Trapezius 140 min [VIP = 1.64, *p*(corr) = 0.75], Pyruvate Trapezius 140–220 min [VIP = 1.62, *p*(corr) = 0.75], PCr [VIP = 1.56, *p*(corr) = −0.72], NTP [VIP = 1.48, *p*(corr) = −0.69], Pyruvate Erector spinae 140–220 min [VIP = 1.11, *p*(corr) = 0.50], and Pi [VIP = 1.11, *p*(corr) = −0.52].

#### 3.3.3 Group differentiation using compounds from saliva, plasma, muscles and body composition variables as regressors

In this step, we regressed group membership using the BC variables and all molecular compounds. The most important significant variables are shown in Table 3 (*R*^2 ^= 0.46, *Q*^2 ^= 0.43, CV-ANOVA *P*-value = 1.25 × 10^−7^). Several of the BC variables were significant in this regression together with five compounds from muscles, three from plasma (IL-2Ra, IL-1-Ra and MDC/CCL22), and four proteins from saliva (IFN-α2α, MIP-1/CCL3, IL-27, and TPO/THPO).

**Table 3:** OPLS-DA of group membership (FM denoted 1 and CON denoted 0) using all body composition (BC) variables (left columns), only non-standardized BC variables (middle columns) and only standardized BC variables (right columns) together with all biochemical variables in saliva, plasma, and muscles as regressors. Variables with VIP > 1.5 are shown.

All BC variables			Non-standardized BC			Standardized BC		
Variables	VIPpred	*p*(corr)	Variables	VIPpred	*p*(corr)	Variables	VIPpred	*p*(corr)
VAT	2.63	0.70	VAT	2.60	0.68	*z*-T-FFMV	2.35	−0.60
*z*-T-FFMV	2.37	−0.62	ASAT	2.29	0.60	M-pyruvate-140 min-Tr	2.30	0.59
ASAT	2.34	0.62	M-pyruvate-140 min-Tr	2.28	0.60	M-pyruvate-140 to 220 min-Tr	2.22	0.58
T-MFI	2.26	0.60	M-pyruvate-140 to 220 min-Tr	2.18	0.58	P-IL-2Ra	2.04	0.53
M-pyruvate-140 min-Tr	2.22	0.59	T-MFI	2.13	0.56	S-IFN-α2a	1.95	0.49
M-pyruvate-140 to 220 min-Tr	2.11	0.57	ES-MFI	2.03	0.53	M-NTP	1.88	−0.49
ES-MFI	2.10	0.55	M-NTP	1.96	−0.52	*z*-VAT	1.86	0.48
*z*-VAT	2.05	0.54	S-IFN-α2a	1.93	0.49	S-MIP-1α/CCL3	1.85	0.46
M-NTP	1.91	−0.51	P-IL-2Ra	1.87	0.50	S-IL-27	1.76	0.44
P-IL-2Ra	1.83	0.49	LF	1.82	0.49	M-PCR	1.75	−0.46
M-PCR	1.80	−0.48	M-PCR	1.82	−0.48	S-TPO/THPO	1.75	0.44
S-IFN-α2a	1.78	0.46	S-MIP-1α/CCL3	1.76	0.44	P-IL-1RA	1.71	0.46
LF	1.76	0.48	P-IL-1RA	1.74	0.48	P-MDC/CCL22	1.66	0.44
S-MIP-1α/CCL3	1.66	0.43	S-IL-27	1.67	0.42	S-FLT3l	1.62	0.41
P-IL-1RA	1.64	0.45	S-TPO/THPO	1.66	0.42	M-pyruvate-140 to 220 min-ES	1.59	0.44
*Z*-ASAT	1.63	0.43	P-MDC/CCL22	1.64	0.45	S-M-CSF	1.51	0.38
P-MDC/CCL22	1.62	0.45	M-pyruvate-140 to 220 min-ES	1.59	0.45	P-ENA-78	1.50	−0.38
S-IL-27	1.57	0.41	S-FLT3l	1.55	0.39			
S-TPO/THPO	1.53	0.40						
M-pyruvate-140 to 220 min-ES	1.52	0.43						
*z*-T-MFI	1.50	0.40						
*R* ^2^	0.46		*R* ^2^	0.42		*R* ^2^	0.42	
*Q* ^2^	0.43		*Q* ^2^	0.37		*Q* ^2^	0.36	
CV-ANOVA *P*-value	1.25 × 10^−7^		CV-ANOVA *P*-value	2.29 × 10^−6^		CV-ANOVA *P*-value	2.47 × 10^−6^	

VIPpred and *p*(corr) are reported for each regressor (i.e., the loading of each variable scaled as a correlation coefficient and therefore standardizing the range from −1 to +1). The sign of *p*(corr) indicates the direction of the correlation with the dependent variable (+, positive correlation (i.e., higher in FM); −, negative correlation (i.e., lower in FM). The three bottom rows report *R*^2^, *Q*^2^, and *P*-value of the CV-ANOVA. P, plasma; S, saliva; M, muscle. For protein names see Supplementary Table 1. VAT, Visceral Adipose Tissue volume; ASAT, Abdominal Subcutaneous Adipose Tissue Volume; T-MFI, Thigh Muscle Fat Infiltration; ES-MFI = Erector Spinae Muscle Fat Infiltration; LF, Liver fat; *z*, standardized body composition variable; PCr, phosphocreatine; NTP, nucleotide triphosphate (NTP, mainly composed of adenosine triphosphate ATP). The concentrations of pyruvate are presented as levels immediately after the trauma period (i.e., at baseline denoted as 140 min) and the mean value of the time points from baseline to the end of the recovery (140–220 min). Tr, trapezius; ES, erector spinae.

Very similar results with respect to the significant biochemical variables from muscles, saliva and plasma were obtained when the regressions included either the non-standardized BC variables or the standardized BC variables (Table 3). In the regression including the non-standardized BC variables VAT, ASAT, and muscle, pyruvate levels of the trapezius were the strongest significant regressors, and in the regression including the standardized BC variables *z*-T-FFMV and the muscle, pyruvate levels of the trapezius were the most important significant regressors (Table 3).

### 3.4 The relationships between clinical variables and compounds from saliva, plasma, and muscles

In Table 4 is given a summary of the significant biochemical regressors of pain intensity, PPT, FIC-index, *z*-VAT and *z*-T-FFMV; details are reported in the Supplementary Tables 6–10.

**Table 4:** An overview of the significant biochemical regressors (marked with X) for the OPLS regressions of pain intensity (NRS), pressure pain thresholds (PPT), FIC-index, *z*-VAT and *z*-T-FFMV. For details of these regressions see Supplementary Material i.e., NRS: Supplementary Table 6, PPT: Supplementary Table 7, FIC-index: Supplementary Table 8, *z*-VAT: Supplementary Table 9 and *z*-T-FFMV: Supplementary Table 10.

Variables	NRS	PPT	FIC-index	*z*-VAT	*z*-T-FFMV
M-Lactate-140 to 220 min-ES				X	
M-NTP		X	X		X
M-PCR	X	X	X		X
M-pyr140-Tr		X	X		X
M-pyruvate-140 to 220 min-ES		X	X		
M-pyruvate-140 to 220 min-Tr		X	X		X
P-ENA-78/CXCL5				X	
P-Eotaxin/CCL11					X
P-Eotaxin-3/CCL26			X		X
P-FLT3l				X	
P-I-309/CCL1		X	X		
P-IFN-α2a				X	X
P-IFN-β	X			X	
P-IFN-*γ*					X
P-IL-12/IL-23p40		X	X		
P-IL-12p70					X
P-IL-16		X	X		
P-IL-17A		X	X		X
P-IL-17B	X				
P-IL-17C	X		X	X	
P-IL-17D	X				
P-IL-17E/IL-25	X				
P-IL-17F	X				
P-IL-18		X	X		X
P-IL-1RA	X	X	X		
P-IL-23	X				
P-IL-29-IFN-L1				X	
P-IL-2Ra		X	X		X
P-IL-3	X			X	
P-IL-31	X				
P-IL-33	X				X
P-IL-6	X	X	X		
P-IL-7		X			X
P-IL-9	X				
P-IP-10			X		
P-MCP-1/CCL2	X	X	X		
P-MCP-3/CCL7				X	
P-MCP-4/CCL13	X		X		
P-M-CSF/CSF-1	X	X	X		
P-MDC/CCL22	X	X	X	X	X
P-MIF		X	X		
P-MIP-1α/CCL3		X	X		
P-MIP-1β/CCL4	X		X		
P-TARC/CCL17	X				
P-TRAIL		X	X	X	
P-TSLP	X		X		
P-VEGF-A	X				X
P-YKL-40		X	X	X	X
S-G-CSF		X			
S-IFN-α2a	X				
S-IL-12-IL-23p40					X
S-IL-15				X	
S-IL-16				X	
S-IL-1α	X				
S-IL-22			X		
S-IL-23					X
S-IL-29-IFN-L1	X		X		
S-IL-6				X	
S-MCP-1/CCL2	X				
S-M-CSF			X		
S-MDC/CCL22					X
S-MIF				X	
S-MIP-5/CCL15				X	
S-TRAIL				X	
S-VEGF-A				X	

In variable names: P, plasma; S, saliva; M, muscle. For protein names see Supplementary Table 1. The concentrations of lactate, and pyruvate are presented as levels immediately after the trauma period—i.e., at baseline (denoted as 140 min) and the mean value of the time points from baseline to the end of the recovery (140–220 min). PCr, phosphocreatine; NTP, nucleotide triphosphate (NTP, mainly composed of adenosine triphosphate ATP); Tr, trapezius; ES, erector spinae.

#### 3.4.1 Pain intensity in FM

As the controls were determined to be pain-free, pain intensity was regressed only for the FM group. The plasma proteins MCP-1/CCL2, MIP-1β/CCL4, TSLP, and IL-1RA and four IL-17 interleukins (B, C, D and F) were the strongest significant regressors and all correlated positively with pain intensity (*R*^2 ^= 0.63, *Q*^2 ^= 0.36, CV-ANOVA *P*-value = 0.033, one predictive component and one orthogonal component) (Supplementary Table 6). Plasma proteins dominated among the significant proteins (Supplementary Table 6).

#### 3.4.2 Pressure pain thresholds

The strongest significant regressors of PPT (*R*^2 ^= 0.33, *Q*^2 ^= 0.29, CV-ANOVA *P*-value = 7.45 × 10^−5^, one predictive component) are shown in Table 4 and in Supplementary Table 7, left part). A mix of muscle and plasma compounds were significant. Since plasma proteins were relatively important in this model, it was possible to regress PPT using only plasma proteins as regressors. This significant model is shown in Table 4 and in Supplementary Table 7, right part) (*R*^2 ^= 0.30, *Q*^2 ^= 0.19, CV-ANOVA *P*-value = 0.006, one predictive component). Low PPT (i.e., hyperalgesia) was associated with high levels of plasma proteins. MDC/CCL22, IL-2Ra, MIF, IL-16, and MCSF/CSF-1 showed the strongest significant correlations with PPT. It was not possible to regress PPT in the two groups separately using compounds from saliva, plasma, and/or muscles.

#### 3.4.3 Psychological distress and health

It was not possible to achieve stable significant regressions of HAD-tot or EQ-VAS in all subjects together or in each of the two subgroups using compounds from saliva, plasma, and muscles.

### 3.5 The relationships between FIC-index and muscle-index and compounds from plasma, saliva, and/or muscles

#### 3.5.1 All subjects

The most significant compounds from plasma, saliva, and/or muscles when regressing FIC-index—i.e., the fat infiltration and content index obtained from the PCA of the non-standardized BC variables (see above) (*R*^2 ^= 0.41, *Q*^2 ^= 0.31, CV-ANOVA *P*-value = 2.75 × 10^−5^; one predictive component—are shown in Table 4 and in Supplementary Table 8, left part). Plasma proteins dominated as significant regressors (Table 4 and Supplementary Table 8, left part).

Also, the regression of FIC-index using only plasma proteins as regressors was significant (Supplementary Table 8, right part) (*R*^2 ^= 0.36, *Q*^2 ^= 0.28, CV-ANOVA *P*-value <0.001; one predictive component). The plasma proteins that were strongest (and positively) associated with FIC-index were M-CSF, MDC, IL-1RA, MIP-1α and IL-2Ra (Supplementary Table 8, right part).

It was not possible to significantly regress FIC-index only using saliva proteins.

The relationship between FIC-index and only the muscle compounds was also analysed (Table 4). The significant OPLS regression (*R*^2 ^= 0.52, *Q*^2 ^= 0.31, CV-ANOVA *P*-value = 9.83 × 10^−5^, one predictive component and one orthogonal component) identified the following compounds as significant: NTP [VIP=1.76, *p*(corr) = −0.62]; PCr [VIP = 1.68, *p*(corr) = −0.58]; Pyruvate Trapezius 140 min [VIP = 1.54, *p*(corr) = 0.53]; Pyruvate Trapezius 140–220 min [VIP = 1.50; *p*(corr) = 0.50]; Pyruvate Erector spinae 140–220 min [VIP = 1.38, *p*(corr) = 0.47]; and muscle pH [VIP = 1.23, *p*(corr) = −0.44].

It was not possible to obtain a significant regression of Muscle-index using compounds from plasma, saliva, and muscles in all subjects.

#### 3.5.2 The two groups separately

No significant models of FIC-index or Muscle-index were obtained from FM or CON when using all compounds as regressors.

### 3.6 The relationships between standardized BC variables and compounds from plasma, saliva, and muscles

As reported above, unlike the non-standardized BC variables, it was not possible to achieve a significant PCA of the standardized BC variables and determine indices. Therefore, we first regressed the four standardized fat infiltration and content variables simultaneously (i.e., four *Y* variables simultaneously—*z*-VAT, *z*-ASAT, *z*-LF and *z*-T-MFI). According to CV-ANOVA, this regression indicated that only *z*-ASAT had significant associations with the compounds. However, it was not possible to significantly regress *z*-VAT in all subjects taken together or in FM. The regression for CON was significant. The significant compounds—a mix of saliva and plasma proteins—are shown in Table 4 and in Supplementary Table 9 (*R*^2 ^= 0.76, *Q*^2 ^= 0.64, CV-ANOVA *P*-value = 7.26 × 10^−6^; one predictive component). Four saliva proteins (IL-6, IL-15, VEGF-A and MIP-5/CCL15) and the plasma protein TRAIL were most strongly correlated with *z*-VAT.

Secondly, we regressed the fifth standardized variable (i.e., *z*-T-FFMV). The regression of the most important substances was significant (*R*^2 ^= 0.45, *Q*^2 ^= 0.31, CV-ANOVA *P*-value = 1.84 × 10^−5^; one predictive component) (Table 4 and Supplementary Table 10). The strongest significant predictors were NTP, PCr, P-IL-7, pyruvate 140 min-Tr, and P-IL-2Ra. No significant regressions were obtained in the two groups of subjects separately.

## 4 Discussion

### 4.1 Major results

In this explorative study we investigated whether FM patients have different profiles regarding inflammatory proteins, metabolites, markers of muscle energetic system, fat infiltration and body composition. Moreover, we explored whether there were significant associations between the specific profiles of biochemical compounds and clinical variables.

After standardizing the body composition measurements to remove the cofounding effect of differences in BMI, we found that fat infiltration and content generally were increased, and fat-free muscle volume of the thigh decreased in FM. The latter variable correlates strongest with intramuscular compounds and with two plasma proteins. In other words, there are differences in fat infiltration and content that cannot be explained by differences in BMI.

Saliva cytokines and chemokines contributed to the significant differentiation between FM and CON, and saliva compounds were increased in FM. When BC variables and muscle compounds were included, a range of variables were significantly associated with FM or CON; fat infiltration and content BC variables together with fat free muscle volume (*z*-T-FFMV) were most important.

Plasma chemokines and cytokines were significantly and positively associated with pain intensity in FM, and negatively with PPT for all subjects taken together. Several of these compounds were also important for the group differentiation and FIC-index. Mainly plasma cytokines and chemokines were significantly and positively associated with the FIC-index in the total sample.

### 4.2 Group differentiation

Our study agrees with two other studies using targeted panels of inflammation-related cytokines and chemokines in FM (74, 75). These three studies together indicate the presence of peripheral low-grade systemic inflammation in FM. In the two earlier studies, plasma samples were analysed (74, 75) using a panel from OLINK Bioscience (Uppsala, Sweden), and the present study used a panel from Meso Scale Discovery (MSD; Maryland, USA). The two panels only partially cover the same proteins. Several of the important plasma proteins found in Bäckryd et al. and Gerdle et al. were identified in saliva in the present study (Table 2)—e.g., MIP-1α/CCL3, MCP-4/CCL13, MIP-3α/CCL20, and GRO-alpha/CXCL1 (74, 75). The findings in these studies and our study partially contradict findings identified in three recent systematic reviews (SR), which are only partially consistent with each other (37–39). One SR reported higher blood levels of IL-6, IL-17A, and IL-4 in FM (37). Another SR reported higher IL-6, IL-8, TNF-α and chemokine eotaxin (CCL11, CCL24, and CCL26) levels and decreased IL-10 in FM (38). The third SR reported lower levels of IL-1β and increased levels of IL-6, IL-8, TNF-α, Interferon-γ, CRP and BDNF (39). The latter SR concluded that there was significant heterogeneity between studies. Several of their identified proteins were found in the present regression (i.e., IL-17A, CCL24 and IL-10) although the latter was increased in this FM cohort (Table 2). These SRs are mainly based on studies investigating a few substances at a time, whereas the present study and Bäckryd et al.'s and Gerdle et al.'s studies are exploratory and based on many cytokines and chemokines in the same arrays (74, 75). Also, explorative proteomic studies of plasma and serum report significant systemic immunity and inflammation alterations in FM compared to healthy controls (33–36).

Specifically, a mix of cytokines and chemokines were important for the differentiation between FM and CON (Table 2). Most of the cytokines were proinflammatory, but there were also increased levels of cytokines generally considered as anti-inflammatory in FM (e.g., IL-10, IL-13, and IL-27). This may reflect mechanisms trying to suppress inflammation in a delicate balance with pro-inflammatory responses. Some cytokines—e.g., IL-27 and IL-10—can exhibit both pro-inflammatory and anti-inflammatory properties depending on the micro-environment (76–78). It may be relevant to briefly relate the five most important saliva proteins (MIP-1α/CCL3, I-TAC/CXCL11, MCP-4/CCL13, MIP-3β/CCL19 and IL-2) to studies concerning nociception and pain. The chemokine *MIP*-*1α/CCL3* has inflammatory properties (79), and elevated blood levels have been reported in chronic widespread pain (mainly FM), lumbar intervertebral disk degeneration, migraine, and prostatitis pelvic pain (75, 80–82). *I*-*TAC/CXCL11* is a proinflammatory chemokine and increased blood levels have been found in FM and in endometriosis patients (79, 83, 84). The functions of *MCP*-*4/CCL13* include secretion of proinflammatory cytokines (85)*;* increased serum levels of *MCP*-*4/CCL13* were found in patients with knee osteoarthritis (OA) and rheumatoid arthritis (RA) (86). *MIP*-*3β/CCL19* both promotes inflammation processes and has a homeostatic function (79, 87); a fourfold increase was found in FM (74). In patients with joint injury and OA, the levels correlated with clinical severity (88). *IL*-*2* is a cytokine that can display both pro- and anti-inflammatory properties (89–92). Increased blood levels of IL-2 have been found in FM according to some studies although some studies have not found altered blood levels of IL-2 (38, 39).

Hence, the available literature including the present study applying targeted (broad panels of cytokines/chemokines) and nontargeted (proteomics) analyses indicate that FM is associated with peripheral low-grade inflammation and immune alterations, and the mechanisms involved cannot be limited to processes in the nociceptive systems. The circulatory system, mouth, and saliva have important roles for immune system host defence mechanisms and tissue homeostasis (93, 94). Nociceptors have bidirectional neuro-immune and neural-vascular interactions (95). At peripheral and central levels, bidirectional signalling pathways exist between the immune and nervous systems (96–101). Top-down mechanisms enable the CNS to influence peripheral inflammatory activity via neuroendocrine and autonomic mechanisms (100, 102). Down-top information on peripheral inflammatory activity is transmitted via peripheral nociceptors and humoral and neuronal pathways to the CNS, which may lead to decreased endogenous pain inhibition, neuroinflammation and sickness behaviour (99, 101, 103–105). In FM/chronic widespread pain, signs of neuroinflammation in CNS have been found (74, 106). During neuroinflammation, the blood-brain barrier endothelium undergoes changes, including increased expression of proinflammatory cytokines and chemokines (107). Blood-brain barrier permeability can alter due to influences from cytokines and complement factors, which facilitates this communication to the CNS (108–110). The importance of recognizing immune alterations for the development and clinical picture of FM is emphasised by results that suggest immune molecules (IgG) from FM patients can transfer fibromyalgia symptoms to mice (111). A longitudinal study of low back pain and temporomandibular disorders in the acute and chronic stages indicate that up-regulation of inflammatory responses in the acute stage protects against chronic development (112). Hence, increased knowledge about the trajectories of the immune system in FM is desirable.

We cannot fully explain why compounds from saliva were more important than compounds from plasma for group differentiation (Table 2). Human saliva is attracting increasing research interest since it reflects local and systemic physiological and pathophysiological conditions together with the low costs for sampling and the non-invasive methodology (113, 114). It is already utilized for diagnostic purposes in certain areas e.g., differential diagnosis of Cushing's syndrome, quantification of 25-hydroxy vitamin D, detection of drug and alcohol abuse (113, 115). Proteomic studies indicate that saliva proteins can differentiate between FM and controls (116, 117). A study of neuropathic pain patients with healthy controls showed that saliva proteins were slightly more important than plasma proteins for the group differentiation (118). Saliva exchanges substances with blood through active carriage, passive diffusion, or facilitated diffusion across the cell membrane (119). Saliva has been characterized as functionally equivalent to serum (120). The salivary glands are regulated by the autonomic nervous system (121). As saliva might partially also reflect central alterations (122, 123), saliva has been used to identify biomarkers of neurodegenerative diseases (124). However, the ability for saliva cytokines and chemokines to differentiate FM from controls needs to be confirmed in other studies.

For the present subjects, we report that both standardized and non-standardized BC can predict group membership. Adding these variables as regressors of group membership increased the explained variation (Table 3). One possibility is that the significance of the BC variables just reflects that patients with FM have more adipose tissue and less fat free muscle volume, without any causal explanation. However, the literature as well as our regression of FIC-index (Table 4 and Supplementary Table 8) show that adipose tissues are indeed active tissues secreting proteins actively involved in immunological and inflammatory processes (46–48). Moreover, most of the identified compounds that correlated with FIC-index have previously been reported as important variables for discriminating FM with obesity from non-obese FM patients (see Supplementary Table 8) (44). Plasma proteomic studies investigating the associations with BMI both in FM/chronic widespread pain and in healthy controls provide further support for this hypothesis (125). Hence, the reasons for the importance of BC variables in the regression (Table 3) may include that they reflect uncovered inflammatory and immune processes. Combining targeted (panels of cytokines and chemokines) and untargeted (e.g., proteomics and metabolomics) methods may increase the knowledge about involved molecular mechanisms.

### 4.3 The relationships between pain intensity and peripheral compounds in FM

The significant regression of pain intensity in FM (Table 4 and Supplementary Table 6) is consistent with plasma and muscle proteomic studies, which have shown significant relationships between proteins and pain intensity in FM and CWP (mainly FM) (35, 125, 126). The plasma proteins MCP-1/CCL2, MIP-1β/CCL4, TSLP, IL-1Ra and four IL-17 interleukins (B, C, D and F) showed the strongest and positive associations with pain intensity (Table 4 and Supplementary Table 6).

Specifically, the chemokine *MCP*-*1/CCL2* is considered proinflammatory (79), but may also have anti-nociceptive functions (76). *MCP*-*1/CCL2* correlated positively with pain intensity in FM (127) and was increased in FM and in chronic neck patients (79, 127–129), although a systematic review of neck pain did not find significant alterations (130). *MIP*-*1β/CCL4*, a proinflammatory chemokine (79), correlated positively with pain intensity in women with work-related neck and/or shoulder pain (131). It is upregulated in several CNS disorders, which may indicate a key role in the neuroinflammatory process (132, 133). *TSLP* has been primarily linked to itching rather than pain behaviour (134). However, serum levels were higher in stage III/IV endometriosis than in controls (135). *IL*-*1Ra* inhibits the activity of IL-1 and therefore has anti-inflammatory properties (136), and serum level was significantly increased in psoriatic arthritis and the levels were associated with joint severity (137). Several members (B, C, D, E/IL-25, and F) of the *IL*-*17* family were important for pain intensity (Table 4 and Supplementary Table 6). IL-17A is the prototypical member of the IL-17 family; the other members are less investigated (138). IL-17 is known for its pro-inflammatory role in autoimmune diseases (138). IL-17 plays a significant role in chronic pain initiation and progression (139), although it also has protective functions (138).

### 4.4 The relationships between pressure pain thresholds and peripheral compounds

The most important plasma proteins negatively correlated with PPT were MDC/CCL22, IL-2Ra, MIF, IL-16, and M-CSF/CSF-1 (Table 4 and Supplementary Table 7, right part). Thus, high concentrations of these proteins were associated with hyperalgesia for pressure. MDC/CCL22 and IL-2Ra were also important regressors of group and pain intensity. *MIF* is mainly a pro-inflammatory cytokine (140), but it also can have a protective role (140). Recently, we reported that MIF from plasma is increased in obese FM patients compared to non-obese FM patients (44). Elevated serum level has been found in chronic back pain (141). *IL*-*16* plays a crucial role in the inflammatory process as it acts as a chemoattractant for peripheral immune cells and has been linked to inflammatory diseases such as asthma, Crohn's disease, and RA (142). Elevated blood level has been found in acute myocardial infarction and in chronic back pain (141, 142). *M*-*CSF/CSF*-*1* has proinflammatory and homeostatic functions (143); high circulating levels has been found in patients with cancer, inflammation, and autoimmune disorders (144).

### 4.5 The relationships between psychological distress and health and peripheral compounds in FM

We found no significant correlations between the peripheral compounds and HAD-tot or EQ-VAS in all subjects together or in each of the two subgroups. Plasma proteomic studies of FM patients have found protein patterns correlating with psychological distress aspects (35, 125). Also, studies using targeted panels of proteins have reported significant corelations between plasma protein patterns and anxiety and depression in a mixed group of chronic pain patients (145). We are not aware of studies investigating the relationships with health. Hence, we have not been able to confirm earlier studies and the reasons for this are unclear.

### 4.6 The relationships between FIC-index and peripheral compounds

Plasma proteins alone could significantly predict FIC-index in all subjects taken together. A mix of increased plasma proteins—i.e., the five most significant were M-CSF/CSF-1, MDC/CCL22, IL-1RA, MIP-1α/CCL3, and IL-2RA—were associated with FIC-index (Table 4 and Supplementary Table 8, right part). These positive associations are consistent with obesity studies reporting activated peripheral immune and inflammation processes (45, 46, 146). For example, VAT and SAT secrete cytokines and chemokines as well as several compounds also found here (Supplementary Table 8): MIP-1α/CCL3, MCP1/CCl2, I-309/CCL1, and IL-18 (46).

The two most important chemokines were MDC/CCL22 and MIP-1α/CCL3 (Supplementary Table 8, right part). The pro-inflammatory chemokine *MDC/CCL22* is increased in serum and in monocytes from FM patients, (83, 147); increased levels were also found in ischemic heart disease (148). *MIP*-*1*α*/CCL3* was also important for group differentiation, *IL*-*1*-*Ra* was a significant regressor of pain intensity in FM, and *M*-*CSF/CSF*-*1* was important for PPT (see above). *IL*-*2Ra* is a receptor for the pro-inflammatory cytokine IL-2, which belongs to the IL-1 family. The IL2-IL-2 receptor pathway is incompletely understood but may be involved in the balance between immunity and tolerance (149).

When muscle compounds were included in the analysis of FIC-index, the explained variation was higher (Supplementary Table 8, left part) than when only using plasma proteins as regressors. NTP and PCr (both negatively associated) and pyruvate levels in trapezius and erector spinae were the most important muscle compounds, although 20 plasma proteins and three saliva proteins were also important (Supplementary Table 8, left part). The causal relationships between the muscle compounds and fat infiltration and content are currently unknown. However, the relationship can potentially be related to physical deconditioning and altered dietary habits in FM.

### 4.7 The relationships between standardized BC variables and peripheral compounds

When standardizing the BC variables to remove the confounding effect of BMI, the group difference in the fat compartment variables became slightly less significant, as expected given the strong link between adipose tissue and body weight and group difference in body weight. Nevertheless, there was still a difference that is beyond what can be explained by body size. That is, the FM group has more adipose tissues than expected in all tissues investigated except the liver. In addition, the standardized thigh muscle volume variable (*z*-T-FFMV) had a stronger group difference than before standardizing, so the FM group had smaller muscles than expected (Table 1).

For *z*-VAT in CON, we found significant associations with compounds from saliva, plasma, and muscle (Table 4 and Supplementary Table 9). Four saliva proteins (IL-6, IL-15, VEGF-A and MIP-5/CCL15) and the plasma protein TRAIL were positively and strongest associated with *z*-VAT (i.e., larger volume of visceral adipose tissue than expected given the subject's BMI). Hence, saliva proteins were more important in this regression, unlike the non-standardized BC variables as indicated by FIC-index (Table 4 and Supplementary Table 8). On the other hand, this regression only considered CON, whereas the regression of FIC-index included all subjects, and no subgroup analyses were significant. Brief descriptions about the most important significant proteins are given in Supplementary text #1.

The standardized fat free muscle volume of the thigh (*z*-T-FFMV) was significantly higher in CON than in FM. The substances that most strongly correlated with *z*-T-FFMV were NTP, PCr, P-IL-7, pyruvate 140 min-Trapezius, and P-IL-2Ra (Table 4 and Supplementary Table 10). Thus, a high *z*-T-FFMV was associated with high levels of NTP and PCr together with low pyruvate levels at baseline. IL-7 promoting, for example, muscle hypertrophy correlated positively, and IL-2Ra correlated negatively with *z*-T-FFMV (150). Both IL-7 and the receptor for IL-2 belong to the IL-2 family, which can promote either cell survival or cell death and modulate differentiation of cells into more terminally differentiated (151). In muscle tissue, the IL-2/IL-2 receptor complex via expansion of regulatory T cells (Treg) mitigate muscle inflammation in situations with muscle repair and regeneration (152). These and earlier results from our group clearly indicate a muscle involvement in FM (19–27). FM patients benefit from physical exercise with respect to important clinical outcomes (28). Future studies may investigate to what extent such improvements are found for *z*-T-FFMV and if such alterations are related to molecular muscle normalizations.

### 4.8 Strength and limitations

The cytokines and chemokines investigated mainly cover proteins at the picomolar levels. The complete understanding of the activated biological processes related to muscle fat infiltration and body fat content in subjects with and without chronic pain may require broader approaches with respect to concentration scales (i.e., from micro- to nano-levels) as well as specific tissue types. Traditional statistical analyses are not advisable with low ratios of subjects to variables (i.e., proteins) as many of the proteins likely are highly intercorrelated. Hence, we applied MVDA, which is a backbone in such situations (71, 153). Moreover, we use a state-of-the art method to remove the confounding effect of BMI for BC measurements. This novel ability to identify actual group differences is a strength of our method.

This study has some limitations, including the relatively small sample size and the cross-sectional design. Moreover, only women were included since FM has a strong female predominance (1–4). Future studies should also include men with FM and employ repeated concentration measurements of proteins. In addition, to understand the initiating factors prospective studies of healthy subjects and patients with acute and local pain conditions are necessary. Research using inflammatory panels and omics studies in FM is still in a descriptive and exploratory stage. In future studies it will be important to identify the involved biological processes; it is less likely that only one or few proteins will be important for group differentiation and for certain aspects of the clinical presentation. Larger studies are clearly required to understand which alterations are common for several diagnoses and which are specific for FM. Here, we focus on the five most important proteins of each regression and their relationships in earlier studies to nociception and pain. When scrutinizing all proteins displayed in Tables 2–4, it is obvious that some proteins (e.g., MIP-1α/CCL3, MDC/CCL22, IFN-α2α, IL-1Ra, and IL-6) are involved in several regressions while other are specific. Within each diagnosis, it will be important to understand the influences of comorbidities as immunological and inflammatory biological processes have been reported for depression and ageing (154–156). In this explorative study we did not standardize the sampling of saliva with respect to the time of the day (157, 158). In future confirmatory studies, standardization is important to reduce the risk of bias (157, 158). Another limitation is that we did not screen the subjects for oral cavity diseases.

### 4.9 Conclusion

In FM, peripheral factors are clearly important for group differentiation, pressure pain threshold, as well as pain intensity. Saliva cytokines and chemokines were elevated in FM. Plasma proteins were correlated with both sensitivity and intensity of pain. In addition, cytokines and chemokines were also clearly associated with muscle fat infiltration and body fat content variables. The standardized fat free muscle volume of the thigh (*z*-T-FFMV) was significantly lower in FM and associated with intramuscular energy metabolites and plasma proteins. FM is clearly associated with the presence of systemic chronic low-grade systemic inflammation, including immune involvement, and one of the main sources for this is peripheral adipose tissue. Therefore, it is reasonable to believe that FM is characterized by a range of interactions between peripheral tissues, such as muscles and the peripheral and central nervous systems, including nociceptive, immune, and neuroendocrine processes. Clearly, future research of FM should focus on complex patterns of molecules and biological processes in several systems.

## Data Availability

The original contributions presented in the study are included in the article/Supplementary Material, further inquiries can be directed to the corresponding author.
