# Supplementary material for: Pain and the biochemistry of fibromyalgia: patterns of peripheral cytokines and chemokines contribute to the differentiation between fibromyalgia and controls and are associated with pain, fat infiltration and content
Source: Front Pain Res (Lausanne). 2024 Jan 18;5:1288024. doi: 10.3389/fpain.2024.1288024 (PMC10830731; doi:10.3389/fpain.2024.1288024)
Supplement: Supplementary file 1 [file Datasheet1.docx]

Supplementary Tables and Text

# Supplementary Tables

**Supplementary Table 1**: Inflammatory protein panel. The panel consisting of 71 inflammatory plasma proteins used in the biochemical analyses. LLOD-ULOD: lower limit of detection – upper limit of detection for each protein in the biochemical analyses. The variation of coefficient (CV) is based on the duplicate run of the standards from high concentration (CV 1) to low concentration (CV 7).

| **Protein ID** | **Protein name** | **ULOD (pg/ml)** | **LLOD (pg/ml)** | **CV 1** | **CV 2** | **CV 3** | **CV 4** | **CV 5** | **CV 6** | **CV 7** |
| --- | --- | --- | --- | --- | --- | --- | --- | --- | --- | --- |
| CTACK/CCL27 | Cutaneous T-cell-attracting chemokine | 3770 | 0.61 | 0.25 | 9.43 | 9.17 | 2.68 | 0.67 | 25.38 | 22.60 |
| ENA78/CXCL5 | Epithelial-derived neutrophil-activating protein 78 | 4070 | 0.12 | 1.18 | 1.27 | 2.35 | 5.03 | 3.95 | 1.93 | 2.51 |
| Eotaxin/CCL11 | Eotaxin | 6150 | 7.10 | 1.11 | 4.33 | 0.49 | 4.28 | 15.49 |  |  |
| Eotaxin2/CCL24 | Eotaxin 2 | 5970 | 2.40 | 1.12 | 5.73 | 10.59 | 8.17 | 7.79 | 9.24 | 18.37 |
| Eotaxin3/CCL26 | Eotaxin 3 | 19700 | 5.44 | 1.31 | 0.32 | 4.14 | 7.90 | 2.62 | 14.79 | 61.32 |
| EPO | Erythropoietin | 50900 | 0.26 | 3.49 | 1.35 | 11.85 | 1.63 | 5.10 | 2.53 | 0.79 |
| FLT3L | Fms-related tyrosine kinase 3 ligand | 6110 | 0.18 | 1.52 | 0.76 | 2.99 | 0.87 | 2.12 | 3.18 | 7.37 |
| Fractalkine | Fractalkine | 94800 | 17.76 | 3.31 | 0.19 | 2.81 | 4.99 | 7.95 | 10.90 | 27.99 |
| GCSF | Granulocyte colony-stimulating factor | 22300 | 1.17 | 1.41 | 1.35 | 2.55 | 2.87 | 5.67 | 2.71 | 6.80 |
| GMCSF | Granulocyte-macrophage colony-stimulating factor | 9570 | 0.03 | 1.15 | 4.37 | 2.82 | 3.94 | 1.39 | 2.45 | 1.86 |
| GROα | Growth-regulated alpha protein | 2400 | 0.07 | 3.59 | 3.30 | 3.46 | 2.14 | 8.33 | 0.07 | 10.45 |
| I309/CCL1 | T lymphocyte-secreted protein I309 | 2320 | 1.75 | 2.88 | 1.37 | 8.90 | 0.38 | 5.54 | 8.10 |  |
| IFNα2a/IFNA2 | Interferon-alpha-2a | 44800 | 0.11 | 0.52 | 2.20 | 1.43 | 0.44 | 6.03 | 2.04 | 1.29 |
| IFNβ | Interferon-beta | 92800 | 1.12 | 0.44 | 1.42 | 1.53 | 3.77 | 0.39 | 0.35 | 3.92 |
| IFNγ | Interferon-gamma | 27400 | 1.75 | 0.22 | 2.56 | 5.26 | 4.80 | 1.80 | 3.43 | 3.13 |
| IL10 | Interleukin-10 | 3610 | 0.02 | 1.89 | 1.33 | 2.10 | 4.22 | 1.93 | 1.14 | 0.98 |
| IL12-IL23p40/IL12B | Interleukin-12/Interleukin-23 p40 | 20600 | 0.37 | 3.70 | 2.12 | 0.02 | 1.67 | 3.12 | 4.33 | 8.03 |
| IL12p70 | Interleukin-12 p70 | 6640 | 0.12 | 2.82 | 2.36 | 0.96 | 3.56 | 2.48 | 1.72 | 0.50 |
| IL13 | Interleukin-13 | 2440 | 1.85 | 0.38 | 2.90 | 1.96 | 2.42 | 26.85 | 11.76 | 83.03 |
| IL15 | Interleukin-15 | 3000 | 0.24 | 2.57 | 1.91 | 1.74 | 4.44 | 7.27 | 2.18 | 8.38 |
| IL16 | Interleukin-16 | 21100 | 1.61 | 9.89 | 2.32 | 6.52 | 5.96 | 9.21 | 2.23 | 2.60 |
| IL17A | Interleukin-17A | 19000 | 0.67 | 6.93 | 5.37 | 8.26 | 4.55 | 3.69 | 3.15 | 5.25 |
| IL17AF | Interleukin-17AF | 14400 | 4.56 | 0.61 | 4.19 | 3.08 | 3.81 | 9.57 | 19.09 | 26.46 |
| IL17B | Interleukin-17B | 3580 | 0.14 | 3.50 | 2.79 | 2.67 | 4.46 | 1.15 | 3.90 | 3.01 |
| IL17C | Interleukin-17C | 20000 | 0.31 | 3.57 | 2.57 | 2.40 | 0.88 | 2.64 | 3.61 | 1.41 |
| IL17D | Interleukin-17D | 45900 | 2.07 | 2.25 | 1.68 | 4.08 | 1.36 | 4.77 | 9.78 | 14.48 |
| IL17E/IL25 | Interleukin-17E/Interleukin-25 | 7720 | 0.24 | 4.38 | 3.64 | 2.94 | 2.74 | 0.38 | 3.94 | 2.02 |
| IL17F | Interleukin-17F | 98800 | 120.99 | 2.20 | 0.64 | 4.44 | 0.44 | 10.18 | 35.57 |  |
| IL18 | Interleukin-18 | 39100 | 0.14 | 2.57 | 1.43 | 2.36 | 2.53 | 4.74 | 3.65 | 0.39 |
| IL1RA/IL1RN | Interleukin-1 receptor antagonist protein | 4630 | 1.81 | 1.55 | 1.34 | 5.17 | 2.22 | 2.46 | 5.51 | 43.41 |
| IL1α | Interleukin-1 alpha | 5800 | 0.14 | 12.69 | 2.33 | 0.06 | 4.12 | 18.65 | 3.06 | 24.33 |
| IL1β | Interleukin-1 beta | 3630 | 1.08 | 5.43 | 0.40 | 4.45 | 2.90 | 3.80 | 0.23 | 0.19 |
| IL2 | Interleukin-2 | 2030 | 0.14 | 0.87 | 2.67 | 2.05 | 0.09 | 2.02 | 6.97 | 4.31 |
| IL21 | Interleukin-21 | 11400 | 1.12 | 3.26 | 4.88 | 0.71 | 4.01 | 0.03 | 39.46 | 124.32 |
| IL22 | Interleukin-22 | 2940 | 0.06 | 2.27 | 4.92 | 2.15 | 3.74 | 4.01 | 3.00 | 4.57 |
| IL23/IL23A | Interleukin-23 | 20900 | 0.41 | 2.99 | 0.16 | 1.29 | 2.44 | 0.87 | 0.44 | 0.67 |
| IL27 | Interleukin-27 | 40100 | 4.06 | 3.05 | 3.39 | 2.88 | 2.54 | 2.77 | 4.53 | 4.66 |
| IL29/IFNL1 | Interleukin-29/Interferon lambda-1 | 8980 | 0.35 | 0.02 | 2.74 | 1.16 | 3.51 | 0.47 | 0.63 | 4.37 |
| IL2Ra | Interleukin-2 receptor subunit alpha | 53100 | 1.55 | 0.71 | 3.57 | 5.25 | 2.66 | 6.37 | 0.72 | 6.69 |
| IL3 | Interleukin-3 | 14700 | 5.37 | 2.91 | 0.13 | 5.43 | 2.09 | 5.35 | 2.03 | 45.36 |
| IL31 | Interleukin-31 | 9470 | 4.56 | 1.30 | 0.66 | 1.18 | 7.68 | 0.42 | 2.87 | 18.10 |
| IL33 | Interleukin-33 | 9030 | 0.11 | 1.41 | 0.95 | 0.04 | 1.20 | 1.06 | 0.85 | 7.95 |
| IL4 | Interleukin-4 | 2040 | 0.02 | 2.38 | 2.11 | 0.03 | 0.52 | 3.05 | 4.09 | 3.52 |
| IL5 | Interleukin-5 | 3910 | 0.08 | 0.20 | 0.97 | 3.66 | 2.00 | 1.24 | 1.28 | 1.21 |
| IL6 | Interleukin-6 | 2060 | 0.06 | 2.86 | 1.11 | 9.57 | 0.94 | 1.80 | 1.34 | 0.32 |
| IL7 | Interleukin-7 | 6570 | 0.17 | 2.77 | 0.19 | 0.64 | 0.56 | 7.62 | 9.74 | 0.42 |
| IL8 | Interleukin-8 | 2010 | 0.03 | 1.50 | 1.21 | 3.33 | 5.43 | 0.21 | 0.08 | 0.15 |
| IL9 | Interleukin-9 | 1490 | 0.03 | 2.27 | 0.08 | 0.94 | 0.01 | 1.03 | 0.84 | 4.91 |
| IP10/CXCL10 | Interferon gamma-induced protein 10 | 9510 | 0.82 | 0.24 | 1.22 | 1.09 | 2.50 | 2.26 | 7.11 | 7.15 |
| ITAC/CXCL11 | Interferon-inducible T-cell alpha chemoattractant | 1960 | 1.31 | 6.40 | 0.22 | 1.14 | 5.61 | 4.08 | 10.34 | 15.32 |
| MCP1/CCL2 | Monocyte chemotactic protein 1 | 5920 | 0.14 | 3.37 | 2.85 | 4.20 | 0.82 | 2.66 | 4.77 | 7.36 |
| MCP2 | Monocyte chemotactic protein 2 | 2160 | 0.03 | 1.87 | 4.14 | 1.17 | 4.22 | 17.26 | 3.22 | 2.81 |
| MCP3/CCL7 | Monocyte chemotactic protein 3 | 4660 | 0.58 | 3.11 | 1.22 | 1.36 | 0.89 | 28.80 | 2.45 | 23.05 |
| MCP4/CCL13 | Monocyte chemotactic protein 4 | 4150 | 1.75 | 0.32 | 2.89 | 0.53 | 2.30 | 5.70 | 6.08 | 12.91 |
| MCSF/CSF-1 | Macrophage colony-stimulating factor 1 | 1960 | 0.06 | 3.93 | 1.60 | 1.97 | 1.09 | 20.79 | 7.14 | 2.82 |
| MDC/CCL22 | Macrophage-derived chemokine | 20100 | 12.83 | 2.57 | 4.70 | 2.21 | 2.47 | 4.50 | 83.10 |  |
| MIF | Macrophage migration inhibitory factor | 27100 | 1.40 | 6.11 | 9.52 | 4.13 | 1.86 | 1.72 | 3.70 | 110.63 |
| MIP1α/CCL3 | Macrophage inflammatory protein 1-alpha | 6000 | 3.64 | 0.71 | 0.52 | 4.01 | 0.16 | 5.48 | 48.83 |  |
| MIP1β/CCL4 | Macrophage inflammatory protein 1-beta | 1860 | 2.58 | 1.38 | 1.80 | 1.99 | 0.86 | 4.00 | 14.95 | 15.62 |
| MIP3α/CCL20 | Macrophage inflammatory protein 3 alpha | 19000 | 0.59 | 2.03 | 3.24 | 1.72 | 1.31 | 4.39 | 0.30 | 1.81 |
| MIP3β/CCL19 | Macrophage inflammatory protein 3 beta | 1900 | 0.64 | 3.51 | 0.21 | 1.44 | 1.25 | 4.43 | 32.61 | 18.48 |
| MIP5/CCL5 | Macrophage inflammatory protein 5 | 30600 | 0.13 | 4.89 | 8.14 | 3.16 | 4.54 | 1.02 | 1.40 |  |
| SDF1α/CXCL12 | Stromal cell-derived factor 1 alpha | 96100 | 75.11 | 1.46 | 3.40 | 1.90 | 0.10 | 0.24 | 2.80 |  |
| TARC/CCL17 | Thymus and activation-regulated chemokine | 2920 | 0.06 | 2.70 | 2.65 | 0.48 | 1.27 | 0.57 | 2.01 | 9.80 |
| TNFα/TNF | Tumour necrosis factor-alpha | 3650 | 0.16 | 2.00 | 2.78 | 5.16 | 4.47 | 3.06 | 2.84 | 4.20 |
| TNFβ/LTA | Tumour necrosis factor-beta | 4170 | 0.13 | 1.86 | 3.20 | 0.70 | 0.91 | 6.42 | 8.15 | 10.06 |
| TPO/THPO | Thrombopoietin | 44800 | 0.87 | 2.71 | 5.56 | 0.72 | 0.68 | 3.10 | 1.94 | 1.30 |
| TRAIL | TNF-related apoptosis-inducing ligand | 11300 | 0.15 | 1.87 | 3.65 | 2.77 | 4.17 | 24.64 | 1.06 | 4.42 |
| TSLP | Thymic stromal lymphopoietin | 8040 | 0.20 | 1.45 | 8.84 | 0.41 | 0.10 | 1.07 | 0.68 | 4.37 |
| VEGFA | Vascular endothelial growth factor A | 5790 | 1.66 | 2.69 | 3.07 | 5.81 | 3.54 | 3.24 | 0.36 | 46.89 |
| YKL40/CHI3L1 | Chitinase-3-like protein 1 | 4860 | 0.14 | 3.05 | 5.75 | 3.70 | 6.62 | 4.19 | 3.19 | 106.86 |

**Supplementary Table 2.** The parameters of the initial regressions. The results are presented as the final regression using variables with VIPpred > 1.5 (see Methods for details)

| Models | R^2^ | Q^2^ | P-value  CV-ANOVA | Predictive components | Orthogonal components | Model |
| --- | --- | --- | --- | --- | --- | --- |
| Group differentiation using compounds from saliva, plasma, and muscles | 0.35 | 0.21 | 0.002 | 1 | 0 | M71 |
| Group differentiation using compounds from saliva | 0.21 | 0.13 | 0.034 | 1 | 0 | M76 |
| Group differentiation using compounds from saliva, plasma, muscles, and all body composition variables | 0.61 | 0.33 | 0.0004 | 1 | 1 | M170 |
| Group differentiation using compounds from saliva, plasma, muscles, and non-standardized body composition variables | 0.61 | 0.31 | 0.0009 | 1 | 1 | M244 |
| Group differentiation using compounds from saliva, plasma, muscles, and standardized body composition variables | 0.61 | 0.27 | 0.0037 | 1 | 1 | M246 |
| Regression of FIC-index using compounds from saliva, plasma, and muscles | 0.60 | 0.21 | 0.040 | 1 | 1 | M181 |
| Regression of FIC-index using compounds from plasma | 0.57 | 0.20 | 0.028 | 1 | 1 | M196 |
| Regression of z-VAT using compounds from saliva, plasma, and muscles in CON | 0.89 | 0.53 | 0.006 | 1 | 1 | M90 |
| Regression of pain intensity using compounds from saliva, plasma, and muscles in FM | 0.74 | 0.01 | n.s. | 1 | 1 | M203 |
| Regression of PPT using compounds from saliva, plasma and muscles | 0.61 | 0.19 | 0.10 | 1 | 0 | M205 |
| Regression of PPT using compounds from plasma | 0.50 | 0.10 | 0.30 | 1 | 1 | M238 |

**Supplementary Table 3.** Loadings from a PCA of the body composition variables (both non-standardized and standardized variables except BMI). The loadings of the most important and intercorrelated variables of each component denoted in bold.

| Variables | p[1] | p[2] |
| --- | --- | --- |
| VAT | **0.39** | 0.23 |
| ASAT | **0.36** | 0.25 |
| T-FFMV | –0.10 | **0.57** |
| T-MFI | **0.40** | 0.01 |
| ES-FFMV | –0.01 | **0.52** |
| ES-MFI | **0.32** | 0.12 |
| LF | 0.25 | 0.22 |
| z-ASAT | **0.27** | –0.04 |
| z-VAT | **0.32** | –0.02 |
| z-LF | 0.08 | –0.15 |
| z-T-MFI | **0.32** | –0.21 |
| z-T-FFMV | -0.30 | **0.39** |

VAT=Visceral Adipose Tissue volume; ASAT=Abdominal Subcutaneous Adipose Tissue volume; T-MFI=Thigh Muscle Fat Infiltration; ES-MFI=Erector Spinae Muscle Fat Infiltration; T-FFMV=Thigh Fat-free Muscle volume; ES-FFMV= Erector Spinae Fat-free Muscle volume; LF=Liver fat; z-= standardized variable.

**Supplementary Table 4.** Loadings from a PCA of the non-standardized body composition variables (except BMI). The loadings of the most important and intercorrelated variables of each component denoted in bold.

| Variables | p[1] | p[2] |
| --- | --- | --- |
| VAT | **0.51** | –0.06 |
| ASAT | **0.48** | −0.02 |
| T-FFMV | 0.11 | **0.68** |
| T-MFI | **0.44** | –0.29 |
| ES-FFMV | 0.17 | **0.66** |
| ES-MFI | **0.39** | –0.12 |
| LF | **0.35** | 0.07 |

VAT: Visceral Adipose Tissue Volume; ASAT: Abdominal Subcutaneous Adipose Tissue Volume; T-MFI: Thigh Muscle Fat Infiltration; ES-MFI: Erector Spinae Muscle Fat Infiltration; T-FFMV: Thigh Fat-free Muscle Volume; ES-FFMV: Erector Spinae Fat-free Muscle Volume; LF: Liver Fat

**Supplementary table 5**. OPLS-DA of group membership (FM denoted 1 and CON denoted 0) using saliva proteins as regressors. Variables with VIPpred > 1.0 are shown.

| Variables | VIP | p(corr) |
| --- | --- | --- |
| S-MIP-1α/CCl3 | 1.57 | 0.84 |
| S-I-TAC/CXCL11 | 1.49 | 0.80 |
| S-MCP-4/CCL13 | 1.46 | 0.78 |
| S-Eotaxin-2/CCL24 | 1.41 | 0.75 |
| S-CTACK/CCL27 | 1.39 | 0.74 |
| S-IL-2 | 1.38 | 0.74 |
| S-MIP-3β/CCL19 | 1.37 | 0.73 |
| S-MIP-5/CCL15 | 1.34 | 0.72 |
| S-MIP-3α/CCL20 | 1.33 | 0.71 |
| S-IL-12p70 | 1.29 | 0.69 |
| S-IL-5 | 1.27 | 0.68 |
| S-MDC/CCL22 | 1.27 | 0.68 |
| S-SDF-1alpha/CXCL12 | 1.26 | 0.67 |
| S-IL-17A-F | 1.25 | 0.67 |
| S-IL-10 | 1.24 | 0.66 |
| S-IL-13 | 1.24 | 0.66 |
| S-IL-17A | 1.23 | 0.66 |
| S-G-CSF | 1.19 | 0.64 |
| S-IL-16 | 1.19 | 0.64 |
| S-MCP-2/CCL8 | 1.18 | 0.63 |
| S-GRO-alpha/CXCL1 | 1.18 | 0.63 |
| S-TNF-β/LTA | 1.18 | 0.63 |
| S-TARC/CCL17 | 1.15 | 0.62 |
| S-EPO | 1.13 | 0.60 |
| S-VEGF-A | 1.11 | 0.60 |
| S-IL-17E-IL-25 | 1.11 | 0.59 |
| S-I-309/CCL1 | 1.10 | 0.59 |
| S-MCP-3/CCL7 | 1.09 | 0.59 |
| S-FLT3L | 1.09 | 0.58 |
| S-IL-15 | 1.09 | 0.58 |
| S-TNF-α | 1.09 | 0.58 |
| S-IL-8 | 1.08 | 0.58 |
| S-IL-4 | 1.08 | 0.58 |
| S-GM-CSF/CSF2 | 1.08 | 0.58 |
| S-Eotaxin-3/CCL26 | 1.06 | 0.57 |
| S-IFN-α2a | 1.06 | 0.57 |
| S-Eotaxin/CCL11 | 1.06 | 0.57 |
| S-MIP-1β/CCL4 | 1.06 | 0.57 |
| S-IL-27 | 1.04 | 0.56 |
| S-TPO/THPO | 1.02 | 0.54 |
| S-M-CSF/CSF1 | 1.01 | 0.54 |
| R^2^ | 0.18 |  |
| Q^2^ | 0.14 |  |
| CV-ANOVA P-value | 0.019 |  |

VIPpred and p(corr) are reported for each regressor (i.e., the loading of each variable scaled as a correlation coefficient and therefore standardizing the range from −1 to +1). The sign of p(corr) indicates the direction of the correlation with the dependent variable (+: positive correlation (i.e., higher in FM); –: negative correlation (i.e., lower in FM). The three bottom rows report R^2^, Q^2^, and P-value of the CV-ANOVA. S: saliva. For protein names, see Supplementary Table 1.

**Supplementary Table 6.** OPLS regression of pain intensity in FM using compounds from saliva, plasma, and muscles as regressors. Variables with VIP > 1.5 are shown.

| Variables | VIPpred | p(corr) |
| --- | --- | --- |
| P-MCP-1/CCL2 | 2.53 | 0.66 |
| P-MIP-1β/CCL4 | 2.35 | 0.59 |
| P-TSLP | 2.26 | 0.54 |
| P-IL-1RA | 2.09 | 0.59 |
| P-IL-17F | 2.09 | 0.51 |
| P-IL-17D | 2.04 | 0.50 |
| P-IL-17C | 1.96 | 0.46 |
| P-IL-17B | 1.88 | 0.45 |
| P-TARC/CCL17 | 1.80 | 0.45 |
| P-IL-3 | 1.78 | 0.43 |
| P-M-CSF/CSF-1 | 1.77 | 0.43 |
| M-PCR | 1.76 | -0.44 |
| P-MDC/CCL22 | 1.75 | 0.45 |
| S-IL-1α | 1.71 | -0.54 |
| P-IL-33 | 1.69 | 0.41 |
| P-IL-17E/IL-25 | 1.68 | 0.41 |
| S-IL-29-IFN-L1 | 1.66 | 0.51 |
| P-IL-23 | 1.65 | 0.39 |
| P-VEGF-A | 1.59 | 0.38 |
| S-IFN-α2a | 1.59 | −0.53 |
| P-IL-6 | 1.59 | 0.42 |
| P-IL-31 | 1.58 | 0.37 |
| S-MCP-1/CCL2 | 1.56 | 0.45 |
| P-IFN-β | 1.54 | 0.36 |
| P-MCP-4/CCL13 | 1.53 | 0.35 |
| P-IL-9 | 1.52 | 0.36 |
| R^2^ | 0.63 |  |
| Q^2^ | 0.36 |  |
| CV-ANOVA P-value | 0.033 |  |

VIP and p(corr) are reported for each regressor (i.e., the loading of each variable scaled as a correlation coefficient and therefore standardizing the range from −1 to +1). The sign of p(corr) indicates the direction of the correlation with the dependent variable (+: positive correlation; ­–: negative correlation). The three bottom rows report R^2^, Q^2^, and P-value of the CV-ANOVA. P: plasma; S: saliva; M: muscle. For protein names, see **Supplementary Table 1**. PCr: phosphocreatine.

**Supplementary Table 7.** OPLS regression of PPT in all subjects taken together using compounds from saliva, plasma, and muscles as regressors (left part) and only plasma compounds as regressors (right part). Variables with VIP > 1.5 are shown.

| Variables | VIPpred | p(corr) | Variables | VIPpred | p(corr) |
| --- | --- | --- | --- | --- | --- |
| M-pyr140-Tr | 2.86 | −0.62 | P-MDC/CCL22 | 2.19 | −0.57 |
| P-IL-1RA | 2.51 | −0.56 | P-IL-2Ra | 2.19 | −0.57 |
| P-IL-16 | 2.45 | −0.53 | P-MIF | 2.06 | −0.54 |
| M-pyruvate-140 to 220 min-Tr | 2.44 | −0.54 | P-IL-16 | 1.94 | −0.51 |
| P-IL-2Ra | 2.35 | −0.51 | P-M-CSF/CSF-1 | 1.93 | −0.50 |
| P-MDC/CCL22 | 2.32 | −0.50 | P-IL-1RA | 1.93 | −0.50 |
| M-NTP | 2.25 | 0.49 | P-TRAIL | 1.84 | −0.48 |
| P-MIF | 2.17 | −0.46 | P-YKL-40 | 1.63 | −0.43 |
| M-PCr | 2.12 | 0.47 | P-IL-7 | 1.59 | 0.42 |
| P-M-CSF/CSF-1 | 2.08 | −0.45 |  |  |  |
| P-YKL-40 | 2.04 | −0.44 |  |  |  |
| P-IL-12/IL-23p40 | 1.94 | −0.42 |  |  |  |
| P-I-309/CCL1 | 1.91 | −0.40 |  |  |  |
| P-MCP-1/CCL2 | 1.90 | −0.42 |  |  |  |
| P-TRAIL | 1.84 | −0.40 |  |  |  |
| M-pyruvate-140 to 220 min-ES | 1.82 | −0.40 |  |  |  |
| P-IL-18 | 1.81 | −0.39 |  |  |  |
| P-IL-6 | 1.67 | −0.36 |  |  |  |
| S-G-CSF | 1.65 | −0.33 |  |  |  |
| P-IL-17A | 1.54 | −0.33 |  |  |  |
| P-MIP-1α/CCL3 | 1.50 | −0.33 |  |  |  |
| R^2^ | 0.33 |  | R^2^ | 0.30 |  |
| Q^2^ | 0.29 |  | Q^2^ | 0.19 |  |
| CV-ANOVA P-value | 7.45e-05 |  | CV-ANOVA P-value | 0.006 |  |

VIP and p(corr) are reported for each regressor (i.e., the loading of each variable scaled as a correlation coefficient and therefore standardizing the range from −1 to +1). The sign of p(corr) indicates the direction of the correlation with the dependent variable (+: positive correlation; ­–: negative correlation). The three bottom rows report R^2^, Q^2^, and P-value of the CV-ANOVA. Tr: trapezius; ES: erector spinae; P: plasma; S: saliva; M: muscle. For protein names see **Supplementary T able 1**. PCr: phosphocreatine; NTP: nucleotide triphosphate (NTP, mainly composed of adenosine triphosphate ATP). The concentrations of pyruvate are presented as mean value of the time points from baseline to the end of the recovery (140–220 min). Tr: trapezius, ES: erector spinae.

**Supplementary Table 8**. OPLS regression of fat infiltration and content index (FIC-index) using the compounds from saliva, plasma, and muscles as regressors (x-variables) (left) and only the plasma proteins as regressors (right) in all subjects together (FM+CON). Variables with VIP > 1.5 are shown. * denotes compound was also important for the differentiation between non-obese and obese FM patients as reported elsewhere [44].

| Variables | VIPpred | p(corr) | Variables | VIPpred | p(corr) |
| --- | --- | --- | --- | --- | --- |
| P-IL-1RA* | 2.45 | 0.60 | P-M-CSF/CSF-1* | 2.17 | 0.60 |
| M-pyruvate-140 to 220 min-Tr* | 2.41 | 0.56 | P-MDC/CCL22* | 2.14 | 0.59 |
| P-MDC/CCL22* | 2.39 | 0.58 | P-IL-1RA* | 2.05 | 0.57 |
| M-pyruvate-140min-Tr* | 2.36 | 0.55 | P-MIP-1α/CCL3* | 1.85 | 0.52 |
| P-M-CSF/CSF-1* | 2.24 | 0.55 | P-IL-2Ra | 1.84 | 0.51 |
| P-MIP-1α/CCL3* | 2.24 | 0.55 | P-MIF | 1.70 | 0.47 |
| P-IL-6* | 2.22 | 0.54 | P-I-309/CCL1 | 1.69 | 0.47 |
| M-NTP | 2.11 | -0.49 | P-MCP-1/CCL2* | 1.66 | 0.46 |
| M-PCr | 2.10 | -0.49 | P-IL-6* | 1.65 | 0.46 |
| P-MCP-1/CCL2* | 2.03 | 0.50 | P-YKL-40 | 1.58 | 0.44 |
| S-IL-29-IFN-L1 | 2.02 | 0.47 | P-IL-12/IL-23p40 | 1.58 | 0.44 |
| P-I-309/CCL1 | 2.00 | 0.48 | P-TRAIL | 1.57 | 0.44 |
| P-IL-12-IL-23p40 | 1.94 | 0.48 | P-MCP-4/CCL13* | 1.55 | 0.43 |
| S-IL-22 | 1.91 | 0.44 |  |  |  |
| P-IL-2Ra | 1.88 | 0.46 |  |  |  |
| P-MCP-4/CCL13* | 1.82 | 0.45 |  |  |  |
| P-MIP-1β/CCL4* | 1.76 | 0.44 |  |  |  |
| M-pyruvate-140 to 220 min-ES* | 1.74 | 0.38 |  |  |  |
| P-IP-10* | 1.74 | 0.43 |  |  |  |
| P-TSLP | 1.70 | 0.42 |  |  |  |
| P-IL-18 | 1.67 | 0.41 |  |  |  |
| P-MIF | 1.65 | 0.39 |  |  |  |
| P-IL-17C* | 1.60 | 0.40 |  |  |  |
| P-IL-16 | 1.59 | 0.39 |  |  |  |
| P-YKL-40 | 1.58 | 0.39 |  |  |  |
| S-M-CSF | 1.57 | 0.36 |  |  |  |
| P-IL-17A | 1.54 | 0.37 |  |  |  |
| P-Eotaxin-3/CCL26 | 1.51 | 0.37 |  |  |  |
| R^2^ | 0.41 |  | R^2^ | 0.36 |  |
| Q^2^ | 0.31 |  | Q^2^ | 0.28 |  |
| CV-ANOVA P-value | 2.75e-05 |  | CV-ANOVA P-value | <0.001 |  |

VIP and p(corr) are reported for each regressor (i.e., the loading of each variable scaled as a correlation coefficient and therefore standardizing the range from −1 to +1). The sign of p(corr) indicates the direction of the correlation with the dependent variable (+: positive correlation; ­–: negative correlation). The three bottom rows report R^2^, Q^2^, and P-value of the CV-ANOVA. P: plasma; S: saliva; M: muscle. For protein names, see Supplementary Table 1. PCr: phosphocreatine; NTP: nucleotide triphosphate (NTP, mainly composed of adenosine triphosphate ATP). Concentrations of pyruvate are presented as levels immediately after the trauma period, i.e., at baseline (denoted as 140 min) and the mean value of the time points from baseline to the end of the recovery (140–220 min). Tr: trapezius; ES: erector spinae.

**Supplementary Table 9**. OPLS of z-VAT in CON using compounds from saliva, plasma, and muscles as regressors. Variables with VIP > 1.5 are shown.

| Variables | VIPpred | p(corr) |
| --- | --- | --- |
| S-IL-6 | 2.69 | 0.60 |
| S-IL-15 | 2.64 | 0.59 |
| S-VEGF-A | 2.60 | 0.57 |
| P-TRAIL | 2.41 | 0.56 |
| S-MIP-5/CCL15 | 2.05 | 0.45 |
| P-IL-29-IFN-L1 | 1.83 | −0.44 |
| P-IFN-β | 1.79 | −0.40 |
| P-YKL-40 | 1.77 | 0.40 |
| P-MCP-3/CCL7 | 1.73 | −0.36 |
| P-IL-17C | 1.66 | −0.33 |
| P-IFN-α2a | 1.65 | −0.39 |
| P-FLT3L | 1.64 | 0.37 |
| S-IL-16 | 1.63 | 0.35 |
| S-TRAIL | 1.63 | 0.36 |
| P-IL-3 | 1.62 | −0.33 |
| S-MIF | 1.58 | 0.35 |
| P-ENA-78/CXCL5 | 1.57 | −0.33 |
| P-MDC/CCL22 | 1.57 | 0.35 |
| M-Lactate-140 to 220 min-ES | 1.55 | −0.33 |
| R^2^ | 0.76 |  |
| Q^2^ | 0.64 |  |
| CV-ANOVA P-value | 7.26e-06 |  |

VIP and p(corr) are reported for each regressor (i.e., the loading of each variable scaled as a correlation coefficient and therefore standardizing the range from −1 to +1). The sign of p(corr) indicates the direction of the correlation with the dependent variable (+: positive correlation; −: negative correlation). The three bottom rows report R^2^, Q^2^, and P-value of the CV-ANOVA. ES: erector spinae; P: plasma; S: saliva; M: muscle. For protein names, see **Supplementary Table 1**. Concentrations of lactate are presented as the mean value of the time points from baseline to the end of the recovery (140–220 min). ES: erector spinae.

**Supplementary Table 10.** OPLS of z-T-FFMV in all subjects using compounds from saliva, plasma, and muscles as regressors. Variables with VIP > 1.5 are shown.

| Variables | VIPpred | p(corr) |
| --- | --- | --- |
| M-NTP | 2.67 | 0.43 |
| M-PCr | 2.64 | 0.44 |
| P-IL-7 | 2.32 | 0.44 |
| M-pyruvate 140 min -Tr | 2.32 | −0.45 |
| P-IL-2Ra | 2.11 | −0.39 |
| P-IL-12p70 | 2.10 | 0.39 |
| P-MDC/CCL22 | 2.09 | −0.39 |
| P-VEGF-A | 2.08 | 0.39 |
| P-IL-33 | 2.01 | 0.37 |
| S-IL-12-IL-23p40 | 1.96 | 0.35 |
| P-Eotaxin-3/CCL26 | 1.96 | −0.36 |
| M-pyruvate-140 to 220 min-Tr | 1.93 | −0.36 |
| P-YKL-40 | 1.81 | −0.35 |
| S-IL-23 | 1.74 | 0.31 |
| P-IL-17A | 1.69 | −0.32 |
| P-Eotaxin/CCL11 | 1.68 | −0.32 |
| P-IL-18 | 1.65 | −0.31 |
| S-MDC/CCL22 | 1.55 | −0.34 |
| P-IFN-α2a | 1.53 | 0.32 |
| P-IFN-γ | 1.51 | 0.29 |
| R^2^ | 0.45 |  |
| Q^2^ | 0.31 |  |
| CV-ANOVA P-value | 1.84e-05 |  |

VIP and p(corr) are reported for each regressor (i.e., the loading of each variable scaled as a correlation coefficient and therefore standardizing the range from −1 to +1). The sign of p(corr) indicates the direction of the correlation with the dependent variable (+: positive correlation; –: negative correlation). The three bottom rows report R^2^, Q^2^, and P-value of the CV-ANOVA. TR: trapezius muscle. P: plasma; S: saliva; M: Muscle. For protein names see **Supplementary Text 1**. PCr: phosphocreatine; NTP: nucleotide triphosphate (NTP, mainly composed of adenosine triphosphate ATP). Concentrations of pyruvate are presented as levels immediately after the trauma period, i.e., at baseline (denoted as 140 min) and the mean value of the time points from baseline to the end of the recovery (140–220 min). Tr: trapezius.

**2 Supplementary Text #1**

***The most important significant proteins in the regression of z-VAT in CON***

Specifically, the myokine IL-6 has both pro- and anti-inflammatory effects [1, 2], exerts regulatory effects in lipid and glucose metabolism and paracrine effects within the muscle [3], and is involved in muscle hypertrophy and myogenesis [4]. Physical inactivity is associated with high systemic levels of IL-6 [3]. During exercise, IL-6 has acute and long-term anti-inflammatory effects and promotes the production of anti-inflammatory cytokines; [3] the long-term effects promote reduction in abdominal adiposity [2]. IL-15, mainly produced in response to pathogens and has regulatory effects on different immune cells, [5] is highly expressed in muscle tissue and affects adipose tissue (e.g., by preventing lipid deposition in pre-adipocytes, increasing muscle glucose uptake, and preventing muscle fibre degradation, i.e., anabolic effects) [2, 5]. Natural killer (NK) cells are important in the initial inflammation events in adipose tissue and can be proliferated and accumulated due to IL-15 production [6]. Obese adipose tissue contains elevated levels of cytokines (e.g., IL-15), which stimulate NK cells [6]. VEGF-A regulates blood vessel growth and especially sprouting angiogenesis and vessel maturation [7, 8]. It has a protective role in adipose tissue and limits obesity.[7] MIP-5/CCL15 appears to be involved in the pathophysiology of some cancers and inflammatory diseases including Type 2 Diabetes [9]. TRAIL – a member of the tumour necrosis factor (TNF) superfamily ­– is upregulated on immune cells during inflammation and plays important roles in inducing apoptosis and in activation-induced cell death (AICID), but it can also induce nonapoptotic pathways [10]. Studies also suggest TRAIL has a protective role in the development of Type 1 and 2 Diabetes [11].

***References***

1. Chen W, Wang L, You W, Shan T. Myokines mediate the cross talk between skeletal muscle and other organs. J Cell Physiol. 2021;236(4):2393-412.

2. Park MJ, Choi KM. Interplay of skeletal muscle and adipose tissue: sarcopenic obesity. Metabolism. 2023;144:155577.

3. Severinsen MCK, Pedersen BK. Muscle-Organ Crosstalk: The Emerging Roles of Myokines. Endocr Rev. 2020;41(4):594-609.

4. Pedersen BK, Febbraio MA. Muscles, exercise and obesity: skeletal muscle as a secretory organ. Nat Rev Endocrinol. 2012;8(8):457-65.

5. Prado G, Sardeli AV, Lord JM, Cavaglieri CR. The effects of ageing, BMI and physical activity on blood IL-15 levels: A systematic review and meta-analyses. Exp Gerontol. 2022;168:111933.

6. Ferno J, Strand K, Mellgren G, Stiglund N, Bjorkstrom NK. Natural Killer Cells as Sensors of Adipose Tissue Stress. Trends Endocrinol Metab. 2020;31(1):3-12.

7. Vliora M, Ravelli C, Grillo E, Corsini M, Flouris AD, Mitola S. The impact of adipokines on vascular networks in adipose tissue. Cytokine Growth Factor Rev. 2023;69:61-72.

8. Ioannidou A, Fisher RM, Hagberg CE. The multifaceted roles of the adipose tissue vasculature. Obes Rev. 2022;23(4):e13403.

9. Zhao B, Zhang M, Xie J, Jiang T, Li J, Yang Z. Implications of increased circulating macrophage inhibitory protein-5 in patients with type 2 diabetes mellitus. Int Immunopharmacol. 2022;109:108916.

10. Cardoso Alves L, Corazza N, Micheau O, Krebs P. The multifaceted role of TRAIL signaling in cancer and immunity. FEBS J. 2021;288(19):5530-54.

11. Koliaki C, Katsilambros N. Repositioning the Role of Tumor Necrosis Factor-Related Apoptosis-Inducing Ligand (TRAIL) on the TRAIL to the Development of Diabetes Mellitus: An Update of Experimental and Clinical Evidence. Int J Mol Sci. 2022;23:3225.
